# Implication of Melanopsin and Trigeminal Neural Pathways in Blue Light Photosensitivity *in vivo*

**DOI:** 10.3389/fnins.2019.00497

**Published:** 2019-05-22

**Authors:** Veronika Marek, Elodie Reboussin, Julie Dégardin-Chicaud, Angéline Charbonnier, Alfredo Domínguez-López, Thierry Villette, Alexandre Denoyer, Christophe Baudouin, Annabelle Réaux-Le Goazigo, Stéphane Mélik Parsadaniantz

**Affiliations:** ^1^R&D, Essilor International, Paris, France; ^2^Sorbonne Université, Institut National de la Santé et de la Recherche Médicale, Centre National de la Recherche Scientifique, Institut de la Vision, Paris, France; ^3^Centre Hospitalier Nationale d’Ophtalmologie des Quinze-Vingts, Paris, France; ^4^CHU Robert Debré, Université Reims Champagne-Ardenne, Reims, France; ^5^Versailles Saint-Quentin-en-Yvelines Université, Versailles, France

**Keywords:** blue light, photophobia, trigeminal pathway, melanopsin, ocular nociception, dry eye, neurotoxicity

## Abstract

Photophobia may arise from various causes and frequently accompanies numerous ocular diseases. In modern highly illuminated world, complaints about greater photosensitivity to blue light increasingly appear. However, the pathophysiology of photophobia is still debated. In the present work, we investigated *in vivo* the role of various neural pathways potentially implicated in blue-light aversion. Moreover, we studied the light-induced neuroinflammatory processes on the ocular surface and in the trigeminal pathways. Adult male C57BL/6J mice were exposed either to blue (400–500 nm) or to yellow (530–710 nm) LED light (3 h, 6 mW/cm^2^). Photosensitivity was measured as the time spent in dark or illuminated parts of the cage. Pharmacological treatments were applied: topical instillation of atropine, pilocarpine or oxybuprocaine, intravitreal injection of lidocaine, norepinephrine or “blocker” of the visual photoreceptor transmission, and intraperitoneal injection of a melanopsin antagonist. Clinical evaluations (ocular surface state, corneal mechanical sensitivity and tear quantity) were performed directly after exposure to light and after 3 days of recovery in standard light conditions. Trigeminal ganglia (TGs), brainstems and retinas were dissected out and conditioned for analyses. Mice demonstrated strong aversion to blue but not to yellow light. The only drug that significantly decreased the blue-light aversion was the intraperitoneally injected melanopsin antagonist. After blue-light exposure, dry-eye-related inflammatory signs were observed, notably after 3 days of recovery. In the retina, we observed the increased immunoreactivity for GFAP, ATF3, and Iba1; these data were corroborated by RT-qPCR. Moreover, retinal visual and non-visual photopigments distribution was altered. In the trigeminal pathway, we detected the increased mRNA expression of cFOS and ATF3 as well as alterations in cytokines’ levels. Thus, the wavelength-dependent light aversion was mainly mediated by melanopsin-containing cells, most likely in the retina. Other potential pathways of light reception were also discussed. The phototoxic message was transmitted to the trigeminal system, inducing both inflammation at the ocular surface and stress in the retina. Further investigations of retina-TG connections are needed.

## Highlights

- Increased photosensitivity is a function of wavelength.
- Blue light aversion is accompanied by clinical signs of dry eye.
- Blue light provokes a response in the trigeminal pathways.
- Intra-retinal melanopsin is the main mediator of blue light photophobia.

## Introduction

Photophobia is a highly debilitating sensory disturbance provoked by visible light (Wu and Hallett, 2017). In patients exposed to normally non-painful illumination, this syndrome causes discomfort and pain in the eye (Digre and Brennan, 2012). One of the most common neurologic disorders that causes photophobia is migraine; indeed, as much as 80% of migraineurs heavily suffer from increased light sensitivity (Albilali and Dilli, 2018). As a result, many studies have already explored the potential mechanisms underlying the light-induced exacerbation of migraine (Noseda et al., 2010, 2016, 2017; Nir et al., 2018). However, symptoms of photophobia are not limited to headache cases. Photophobia in general and greater sensitivity to blue light in particular are common for many ophthalmological (dry eye, blepharitis, retinal dystrophy), neurological (blepharospasm, traumatic brain injury) and even psychiatric (depression, anxiety) disorders (Digre and Brennan, 2012). Nonetheless, so far, there have been no major randomized control trials for photophobia management (Albilali and Dilli, 2018). The current treatment of this disorder actually remains a challenge for ophthalmologists and relies primarily on optical means such as wearing filtering glasses (Hoggan et al., 2016; Katz and Digre, 2016). Ubiquitous presence of artificial light sources highly emitting in blue spectrum complicates the situation additionally (Lupis, 2017; Text Request, 2017).

Several hypotheses about the potential origin of light-aversive behavior have been proposed (Digre and Brennan, 2012; Wu and Hallett, 2017) appealing to the roles of the retina (Dolgonos et al., 2011; Matynia et al., 2012, 2015), trigeminal nerves and neighboring blood vessels (Okamoto et al., 2009, 2011, 2012; Rahman et al., 2015; Matynia et al., 2016). Nonetheless, our understanding of photophobia process is still elusive and much of its neurochemistry remains unknown. In the current work, we used behavior tests and various pharmacological treatments to investigate *in vivo* which neurological circuits might be implicated in blue-light aversion.

Photophobia is definitely linked to inflammation and pain sensation; however, a pathway for light as a stress-related nociceptive stimulus remains unclear (Digre and Brennan, 2012; Wu and Hallett, 2017). We already demonstrated *in vivo* the implication of peripheral and central neuro-inflammatory processes in pain-associated ocular damage (Launay et al., 2016). Moreover, we recently reported *in vitro* the phototoxicity of blue light in epithelial cells of ocular surface (Marek et al., 2018). Both studies were performed within the scope of dry eye disease whose sufferers frequently complain of higher daily photosensitivity (Wade, 2015; Stapleton et al., 2017). Hence, in the present work, we investigated clinically the inflammatory signs induced at the ocular surface by exposure to blue light. We also analyzed the neural phototoxic processes that accompanied the blue-light photophobia.

## Results

### Blue Light Aversion Is Accompanied by Inflammation in the Lacrimal Functional Unit

**FIGURE 1:**
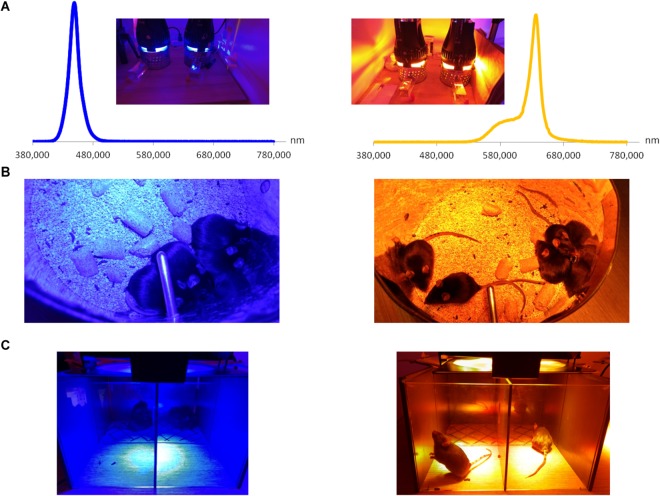
Custom-mounted illumination system. **(A)** Illumination system and relative spectra of LED sources. **(B)** When placed together (and not in separate compartments), mice exposed to blue illumination demonstrated light aversion by hiding behind each other; such behavior did not take place for yellow exposure. **(C)** Behavior test: mice are placed in half-illuminated boxes and allowed to move freely. As in the previous figure, mice exposed to blue illumination demonstrated a strong light aversion, as compared to the yellow one under which animals preferred to stay.

**FIGURE 2:**
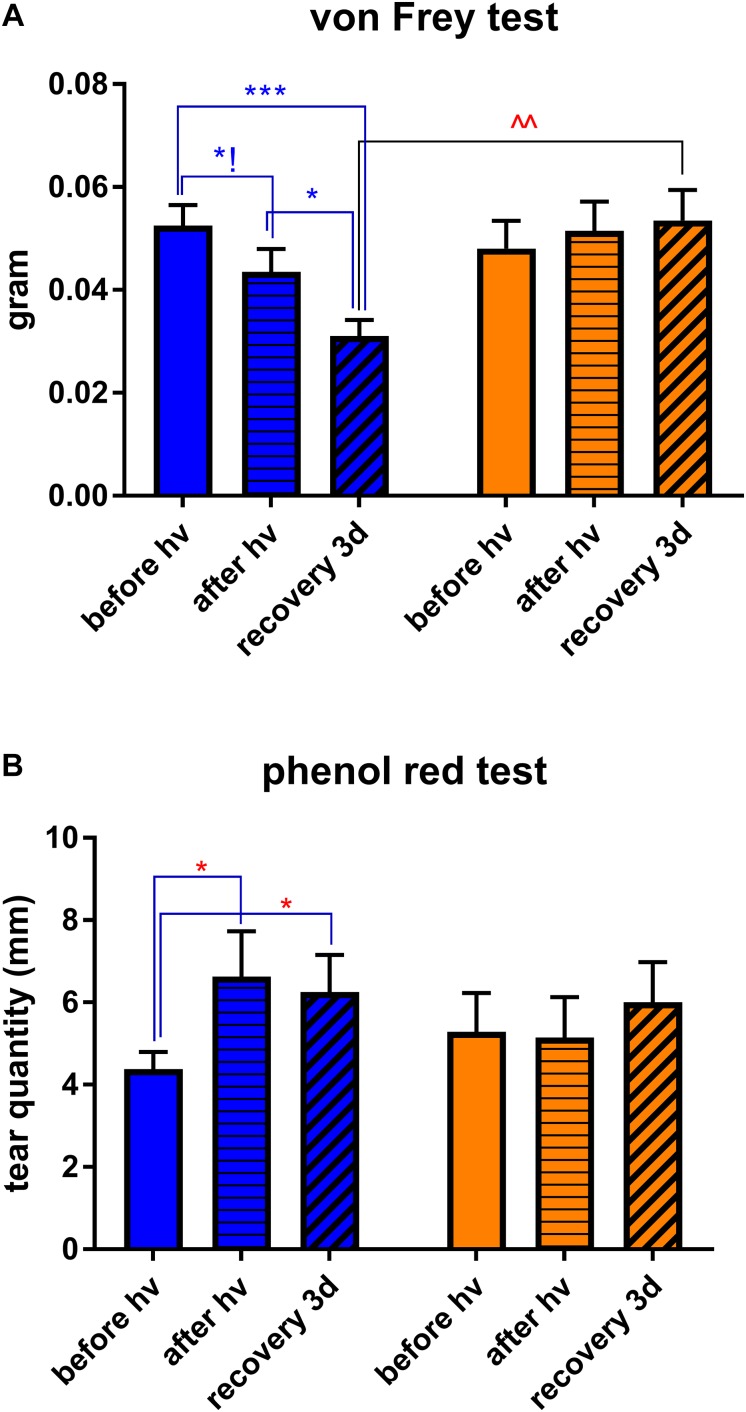
Clinical assessments. Measurements were made at three time points: *before hν*, before the beginning of illumination; *after hν*, directly after 3 h of illumination; *recovery 3d*, after 3 days of recovery in standard illumination conditions of animal unit. **(A)** Measurement of corneal mechanical sensitivity performed by means of von Frey hair test. Greater values mean lower corneal sensitivity. Statistical significance: *blue illumination* group: before hν vs. after hν – *q* = 0.0361, *p* = 0.1031; before hν vs. recovery 3d – *q* = 0.0003, *p* = 0.003; after hν vs. recovery 3d – *q* = 0.0136, *p* = 0.0260; *recovery 3d* group: blue vs. yellow – *q* = 0.0041, *p* = 0.0020. **(B)** Measurement of tear quantity performed by means of phenol red thread test placed into the eye for 30 s. Greater distances mean more important lacrimation. Statistical significance for the *blue illumination* group: before hν vs. after hν – *q* = 0.0403, *p* = 0.0192; before hν vs. recovery 3d – *q* = 0.0498, *p* = 0.0475. Blue and yellow bars correspond to blue and yellow exposures, respectively. All the data are presented as mean ± SEM. Differences were considered significant when *p* < 0.05 (^∗^/^∧^), *p* < 0.01 (^∗∗^/^∧∧^), *p* < 0.001 (^∗∗∗^/^∧∧∧^) or *p* < 0.0001 (^∗∗∗∗^/^∧∧∧∧^). Stars correspond to comparisons between values at different time points, within one spectrum. Carets correspond to comparison between blue-illuminated and yellow-illuminated mice, at the same time point. Red color means increase and blue color decrease in values.

In our preliminary experiments, we put four mice in mirrored-wall boxes exposed to light and let them freely move and interact with each other during all the 3 h of illumination. Mice exposed to blue spectrum exhibited strong aversion to light and permanently hid one behind another. Control yellow-illuminated mice did not demonstrate such kind of behavior (Figure 1B). To eliminate the inter-animal interactions, mice were then placed in individual compartments and assessed clinically, either directly after the end of 3-h exposure or after 3 days of recovery in standard lighting conditions, since it was reported that blue-light-induced inflammation was present after a recovery period (Krigel et al., 2016). We set the recovery time to 3 days because Feng et al. (2017) observed the peak for various inflammatory biomarkers at this time point. Blue light provoked a significant increase in corneal mechanical sensitivity (von Frey hair test) compared to baseline (before-illumination value). After the recovery time, this result only deteriorated: the correspondent value was significantly different from the one of yellow-illuminated mice (Figure 2A). Moreover, blue-light-exposed mice demonstrated a significant increase in tear volume either directly after illumination or after the recovery period (Figure 2B). These signs were not observed in mice exposed to yellow light.

**FIGURE 3:**
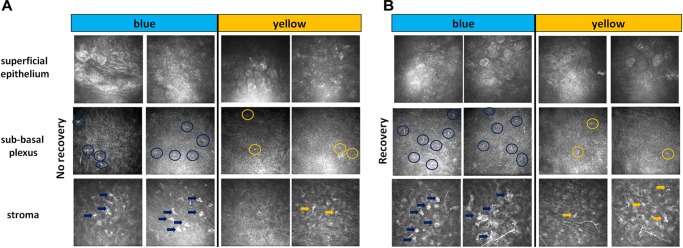
IVCM results. Representative images of non-invasive IVCM examination performed directly after exposure to light (**A**, *no recovery*) or after 3 days of recovery in standard illumination conditions of animal unit (**B**, *recovery*). Alterations were observed in the three following corneal layers: superficial epithelium (cell nuclei in blue-illuminated mice became more hyperreflective), sub-basal plexus (dendritic cells are marked by circles) and stroma (activated keratocytes are marked by arrows).

The slit-lamp examination did not reveal any noticeable differences in fluorescein staining, i.e., no corneal epithelial damage (data not shown). We then used IVCM to explore all the layers of the cornea: epithelium, sub-basal plexus, stroma and endothelium. Directly after light exposure, we observed a slight activation of cells in superficial epithelium (hyperreflective nuclei), some dendritic cells in sub-basal plexus and activated keratocytes in stroma. These inflammatory signs were more pronounced after the exposure to blue light as compared to the yellow one (Figure 3A). After 3 days of recovery, the clinical inflammatory signs decreased or disappeared for the yellow light while they significantly intensified for the blue light (Figure 3B). The corneal endothelium was not damaged whatever the conditions (data not shown).

### Role of the Retina in Blue Light Phototoxicity and Aversion

**FIGURE 4:**
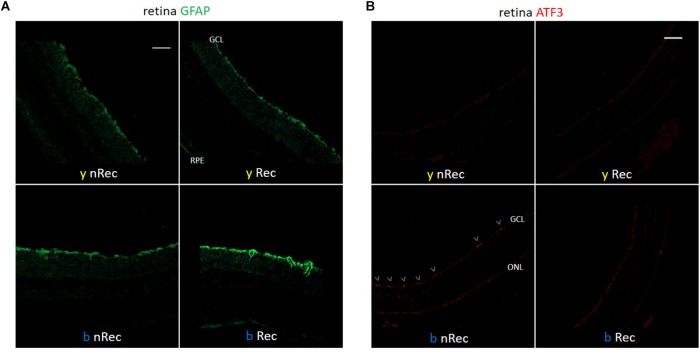
Light-induced retinal inflammation (1). Immunohistochemistry was performed on the retinas of blue- (*b*) and yellow-exposed (*y*) mice either immediately after illumination (*nRec*) or in 3 days of recovery (*Rec*). Results of anti-GFAP **(A)** and anti-ATF3 (**B**, immuno-activated cells are marked by arrowheads) stainings are presented. Magnification is 10×**(A,B)**, scale bars correspond to 100 μm.

**FIGURE 5:**
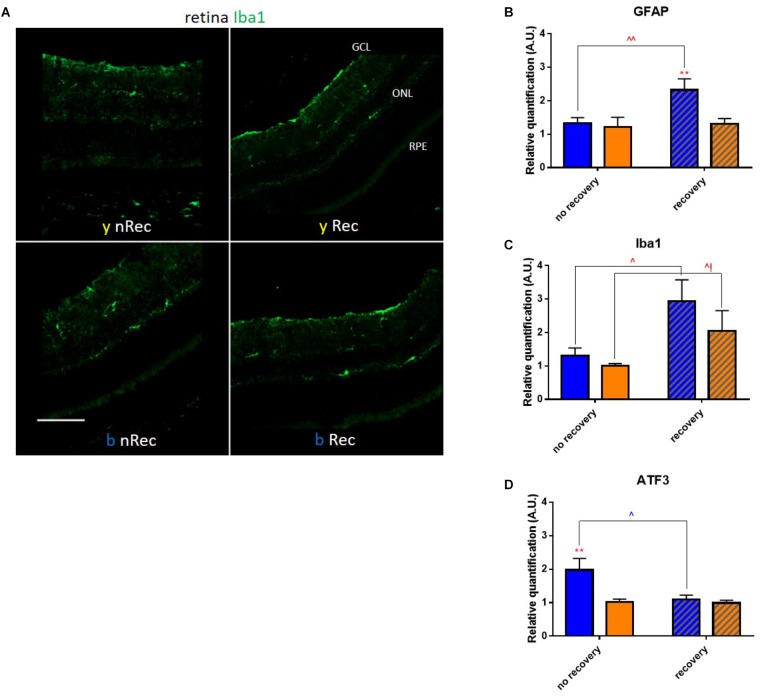
Light-induced retinal inflammation (2). **(A)** Immunohistochemistry was performed on the retinas of blue- (*b*) and yellow-exposed (*y*) mice either immediately after illumination (*nRec*) or in 3 days of recovery (*Rec*). Results of anti-Iba1 staining is presented. Magnification is 20x, scale bar corresponds to 100 μm. **(B–D)** Results of RT-qPCR analysis on the retinas: mRNA expression of GFAP (B), ATF3 (C) and Iba1 (D). Statistical significance: - GFAP: *blue* no recovery vs. recovery – *q* = 0.0101, *p* = 0.0096; *recovery* blue vs. yellow – *q* = 0.0074, *p* = 0.0070; - ATF3: *blue* no recovery vs. recovery – *q* = 0.0230, *p* = 0.0219; *no recovery* blue vs. yellow – *q* = 0.0057, *p* = 0.0054; - Iba1: *blue* no recovery vs. recovery – *q* = 0.0118, *p* = 0.0113; *yellow* no recovery vs. recovery – *q* = 0.0428, *p* = 0.0814. Blue and yellow bars correspond to blue and yellow exposures, respectively; clear and hatched bars correspond to the time points of mice dissection, either directly after illumination (*no recovery*) or in 3 days of recovery (*recovery*), respectively. All the data are presented as mean ± SEM. Differences were considered significant when *p* < 0.05 (^∗^/^∧^), *p* < 0.01 (^∗∗^/^∧∧^), *p* < 0.001 (^∗∗∗^/^∧∧∧^) or *p* < 0.0001 (^∗∗∗∗^/^∧∧∧∧^). Stars correspond to comparisons between blue-illuminated and yellow-illuminated mice, within one recovery or non-recovery group. Carets correspond to comparison of mice assessed directly after illumination to the ones assessed after 3 days of recovery, within the same spectra. Red color means increase and blue color decrease in values.

Retina is the most well-known light signal receiver; it may therefore be implicated as the first mediator of phototoxicity. In 3 days after exposure to blue light, we observed the activation of GFAP dendritiform cells, much more pronounced than directly after the end of illumination (Figure 4A). On the contrary, the ATF3 immunostaining revealed an important fluorescent signal directly after exposure but not after the recovery period (Figure 4B). The signals were much weaker or absent in yellow-illuminated mice. After 3 days of recovery, Iba1 immunolabeling showed an increased inflammatory reaction for both spectra with a slightly greater staining for the blue one (Figure 5A). These results were confirmed by the qPCR analysis (Figure 5B–D).

**FIGURE 6:**
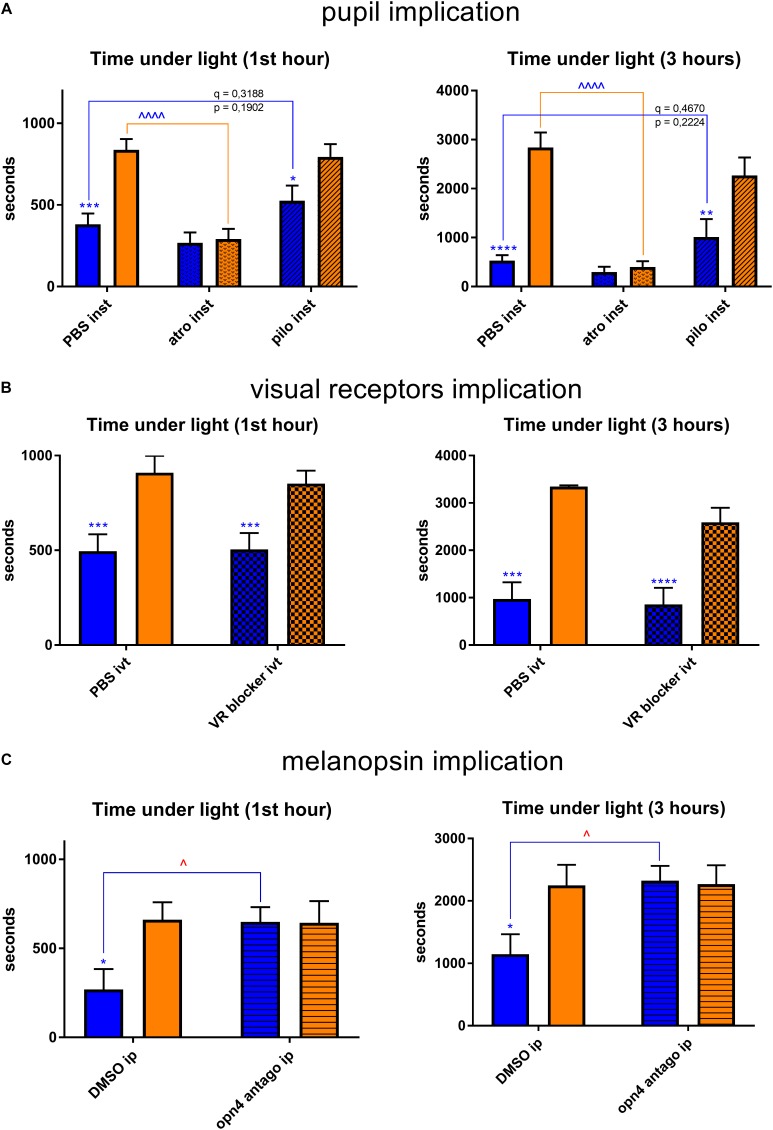
Retina-related behavioral tests. Graphs illustrate the time spent in the illuminated part of the box during the chosen representative periods. For more detail, see “Materials and Methods” section. **(A)** Pupils were dilated with atropine (*atro*) or constricted with pilocarpine (*pilo*). One drop per eye was instilled (*inst*) bilaterally 5 min before the start of light exposure (1st hour: *PBS* blue vs. yellow – *q* = 0.0004, *p* = 0.0003; *pilo* blue vs. yellow – *q* = 0.0057, *p* = 0.0108; *yellow* PBS vs. atro – *q* < 0.0001, *p* < 0.0001; 3 h: *PBS* blue vs. yellow – *q* < 0.0001, *p* < 0.0001; *pilo* blue vs. yellow – *q* = 0.0006, *p* = 0.0012; *yellow* PBS vs. atro – *q* < 0.0001, *p* < 0.0001). **(B)** Visual receptors’ pathway was blocked (*VR blocker*). 2 μL of drug (the composition is described in “Materials and Methods” section) was injected intravitreally (*ivt*) bilaterally 5 min before the start of light exposure (1st hour: *PBS* blue vs. yellow – *q* = 0.0006, *p* = 0.0012; *VR blocker* blue vs. yellow – *q* = 0.0090, *p* = 0.0086; 3 h: *PBS* blue vs. yellow – *q* < 0.0001, *p* < 0.0001; *VR blocker* blue vs. yellow – *q* = 0.0003, *p* = 0.0002). **(C)** Melanopsin antagonist was injected (*opn4 antago*) intraperitoneally (*ip*, 30 mg/kg) 15 min before the start of light exposure (1st hour: *blue* DMSO vs. opn4 antago – *q* = 0.0223, *p* = 0.0212; *DMSO* blue vs. yellow – *q* = 0.0123, *p* = 0.0117; 3 h: *blue* DMSO vs. opn4 antago – *q* = 0.0155, *p* = 0.0147; *DMSO* blue vs. yellow – *q* = 0.0128, *p* = 0.0122). Blue and yellow bars correspond to blue and yellow exposures, respectively; clear bars and hatched bars correspond to animals with control (vehicle – PBS or DMSO) or specific drug treatments, respectively. All the data are presented as mean ± SEM. Differences were considered significant when *p* < 0.05 (^∗^/^∧^), *p* < 0.01 (^∗∗^/^∧∧^), *p* < 0.001 (^∗∗∗^/^∧∧∧^) or *p* < 0.0001 (^∗∗∗∗^/^∧∧∧∧^). Stars correspond to comparisons between blue-illuminated and yellow-illuminated mice, treated with the same drug. Carets correspond to comparisons between control and drug-treated animals. Red color means increase and blue color decrease in values. For the results close to be significant, correspondent *p-* and *q*-values are marked on the graph.

We then supposed that blue-light aversion may depend on the luminous flux that reached the retina. Therefore, we performed behavioral tests in which we compared the blue-light aversion between mice instilled (*inst*) with atropine (*atro*) for pupil dilatation (to increase retinal illumination), with pilocarpine (*pilo*) for pupil constriction (to decrease retinal illumination) and with PBS for the control condition. We also tested the instillation of NaCl as control condition and found no significant difference with PBS instillation (data not shown). Pupil dilatation induced yellow-light aversion that was not observed in our previous experiments. Pupil constriction did not change the behavior under yellow light. It provided with a small trend for a decrease in blue-light aversion; however, this trend appeared to be far from statistically significant (*q* = 0.3188, *p* = 0.1902 after 1 h of exposure; *q* = 0.4670, *p* = 0.2224 after 3 h of exposure). Thus, the blue-light aversion was always present and did not exhibit any significant changes due to pupil size alterations (Figure 6A).

**FIGURE 7:**
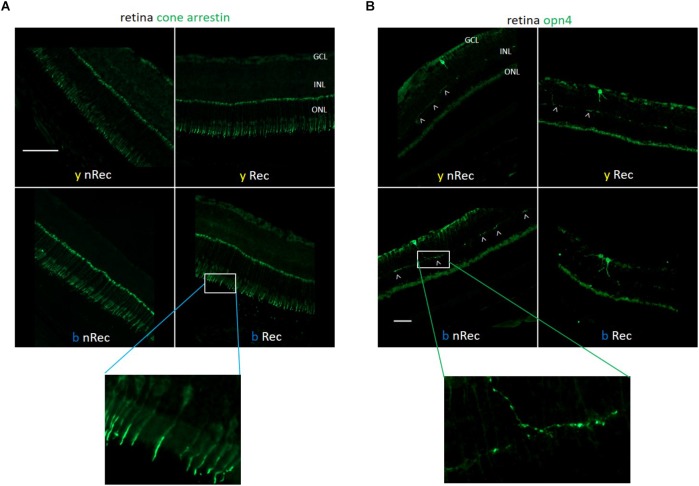
Role of retinal photoreceptors (1). Immunohistochemistry was performed on the retinas of blue- (*b*) and yellow-exposed (*y*) mice either immediately after illumination (*nRec*) or in 3 days of recovery (*Rec*). Results of anti-Cone Arrestin **(A)** and anti-opn4 **(B)** stainings are presented. Insets with higher zoom are provided. Magnification is 20×**(A)** and 10×**(B)**, scale bars correspond to 100 μm.

**FIGURE 8:**
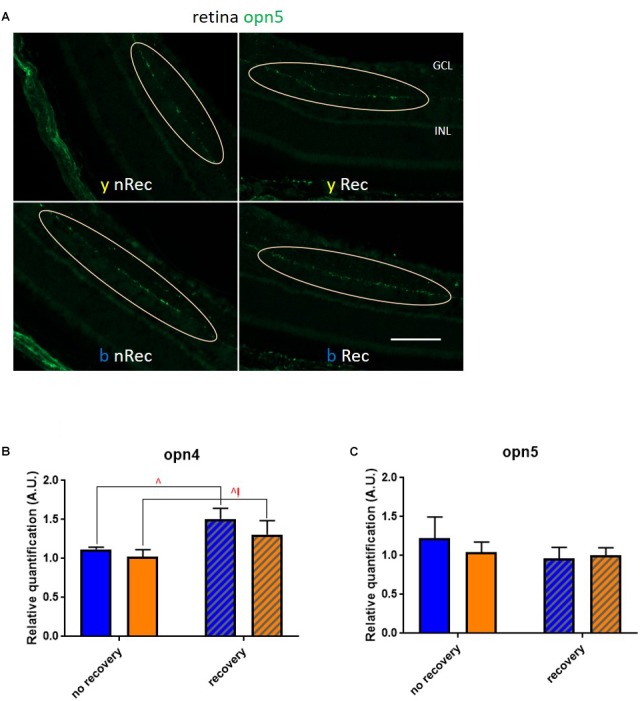
Role of retinal photoreceptors (2). **(A)** Immunohistochemistry was performed on the retinas of blue- (*b*) and yellow-exposed (*y*) mice either immediately after illumination (*nRec*) or in 3 days of recovery (*Rec*). Results of anti-opn5 (**C**, localization of neuropsin-expressing cells is circled) staining is presented. Magnification is 20×, scale bars corresponds to 100 μm. **(B,C)** Results of RT-qPCR analysis on the retinas: mRNA expression of opn4 (**B**; *blue* no recovery vs. recovery – *q* = 0.0174, *p* = 0.0166; *yellow* no recovery vs. recovery – *q* = 0.0499, *p* = 0.0951) and opn5 **(C)**. Blue and yellow bars correspond to blue and yellow exposures, respectively; clear and hatched bars correspond to the time points of dissection, either directly after illumination (*no recovery*) or in 3 days of recovery (*recovery*), respectively. All data are presented as mean ± SEM. Differences were considered significant when *p* < 0.05 (^∗^/^∧^), *p* < 0.01 (^∗∗^/^∧∧^), *p* < 0.001 (^∗∗∗^/^∧∧∧^) or *p* < 0.0001 (^∗∗∗∗^/^∧∧∧∧^). Carets correspond to comparison of mice assessed directly after illumination to the ones assessed after 3 days of recovery, within the same spectra. Red color means increase and blue color decrease in values.

Next, we investigated the role of retinal light receptors. According to our immunohistochemistry study, the rod layer did not exhibit noticeable differences either between two spectra or between two time points of assessment (before and under recovery) (data not shown). However, for the blue light, we observed numerous “holes” in the cone layer while for the yellow exposure it was almost untouched (Figure 7A). To evaluate the status of non-visual light receptors, we performed stainings with anti-melanopsin (anti-opn4) and anti-neuropsin (anti-opn5) antibodies. For both illuminations, we observed a new pattern of melanopsin location: after the recovery time, the signal was less present in axons and accumulated more in cell bodies (Figure 7B). RT-qPCR analysis revealed an increase in melanopsin mRNA expression after recovery for both spectra (Figure 8B). The neuropsin exhibited no significant changes either in immunohistological or in RT-qPCR studies (Figure 8A,C).

Taking into account these findings, we further performed the behavioral tests to investigate whether light aversion would change if we disrupted retinal light reception or processing. We verified, by measuring the optokinetic response, that mice in which retinal visual receptors (*VR*) pathway was blocked (for both rods and cones, see “Materials and Methods” section for the details) had no significant visual responses (Supplementary Figure S1). We found that injection with the correspondent drug (*VR blocker*) did not alter the behavior of mice at any illumination (Figure 6B). However, intraperitoneal (*ip*) injection of melanopsin antagonist (*opn4 antago*) did significantly decrease the blue-light aversion (Figure 6C).

### Implication of Out-Retinal Melanopsin and Trigeminal Pathways

**FIGURE 9:**
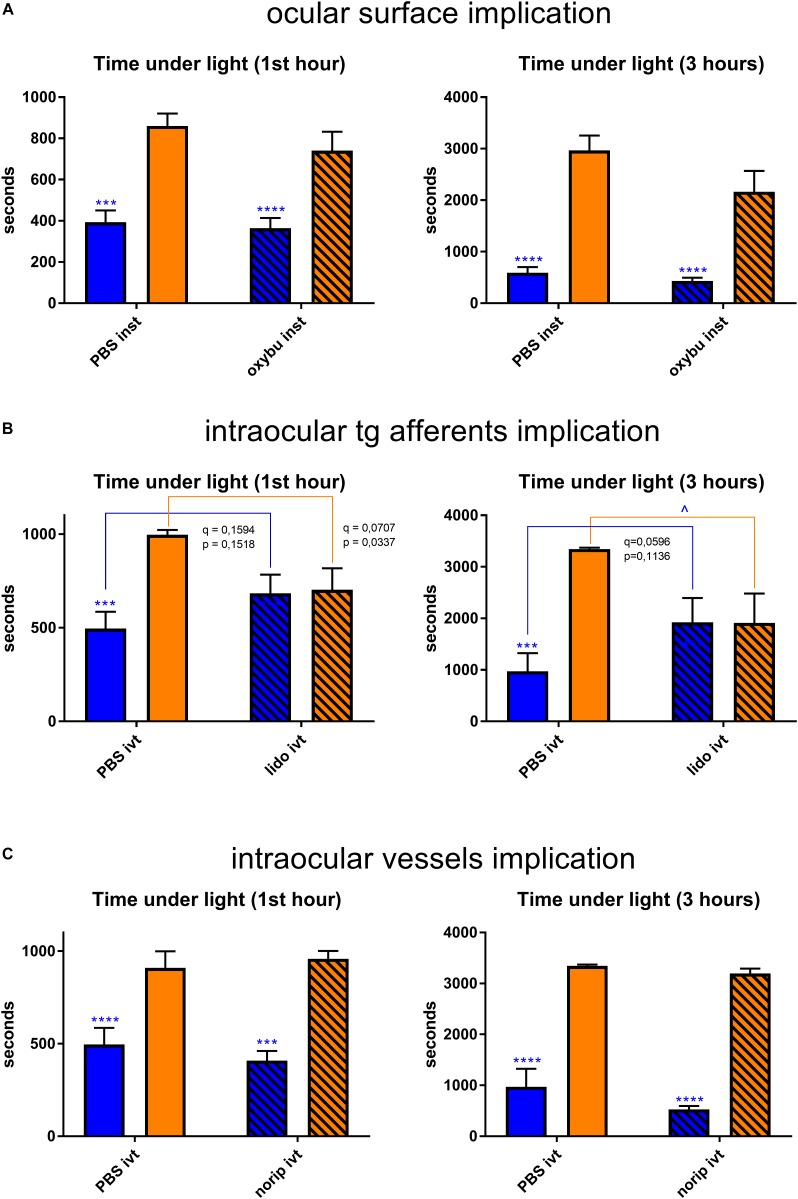
Trigeminal pathway-related behavioral tests. Graphs illustrate the time spent in the illuminated part of the box during the chosen representative periods. For more detail, see “Materials and Methods” section. **(A)** Ocular surface was anesthetized with oxybuprocaine (*oxybu*). One drop per eye was instilled (*inst*) bilaterally directly before the start of light exposure (1st hour: *PBS* blue vs. yellow – *q* = 0.0008, *p* = 0.0008; *oxybu* blue vs. yellow – *q* < 0.0001, *p* < 0.0001; 3 h: *PBS* blue vs. yellow – *q* < 0.0001, *p* < 0.0001; *oxybu* blue vs. yellow – *q* = 0.0001, *p* = 0.0001). **(B)** Intraocular trigeminal afferents were anesthetized with lidocaine (*lido*). 2 μL of drug was injected intravitreally (*ivt*) bilaterally 5 min before the start of light exposure (1st hour: *PBS* blue vs. yellow – *q* = 0.0007, *p* = 0.0006; 3 h: *yellow* PBS vs. lido – *q* = 0.0210, *p* = 0.0200; *PBS* blue vs. yellow – *q* = 0.0004, *p* = 0.0003). **(C)** Intraocular blood vessels were constricted with norepinephrine (*norip*). 2 μL of drug was injected intravitreally (*ivt*) bilaterally 5 min before the start of light exposure (1st hour: *PBS* blue vs. yellow – *q* = 0.0003, *p* = 0.0003; *norip* blue vs. yellow – *q* < 0.0001, *p* < 0.0001; 3 h: *PBS* blue vs. yellow – *q* < 0.0001, *p* < 0.0001; *norip* blue vs. yellow – *q* < 0.0001, *p* < 0.0001). Blue and yellow bars correspond to blue and yellow exposures, respectively; clear bars and hatched bars correspond to animals with control (vehicle – PBS) or specific drug treatments, respectively. All the data are presented as mean ± SEM. Differences were considered significant when *p* < 0.05 (^∗^/^∧^), *p* < 0.01 (^∗∗^/^∧∧^), *p* < 0.001 (^∗∗∗^/^∧∧∧^) or *p* < 0.0001 (^∗∗∗∗^/^∧∧∧∧^). Stars correspond to comparisons between blue-illuminated and yellow-illuminated mice, treated with the same drug. Carets correspond to comparisons between control and drug-treated animals. Red color means increase and blue color decrease in values. For the results close to be significant, correspondent *p*- and *q*-values are marked on the graph.

Non-retinal tissues that potentially contain melanopsin were then studied. Topical instillation (*inst*) of local anesthetic (oxybuprocaine – *oxybu*) on the cornea did not exhibit any impact on behavior under light (Figure 9A). Intravitreal (*ivt*) injection of lidocaine (*lido*), which silenced all the probable trigeminal afferents reaching the choroid and the retina, provided with a trend toward a decrease of blue-light aversion; however, it appeared to be non-significant (*q* = 0.1983, *p* = 0.1888 after 1 h of exposure; *p* = 0.0596, *q* = 0.1136 after 3 h of exposure; Figure 9B). Surprisingly, lidocaine ivt injection significantly decreased the time that mice spent under yellow light. Another possibility for light aversion circuit would be to transmit the phototoxic message from the retina to trigeminal afferents situated near blood vessels by dilatation of the latter. However, the ivt injection of norepinephrine (a vasoconstrictor – *norip*) did not impact mice behavior (Figure 9C).

**FIGURE 10:**
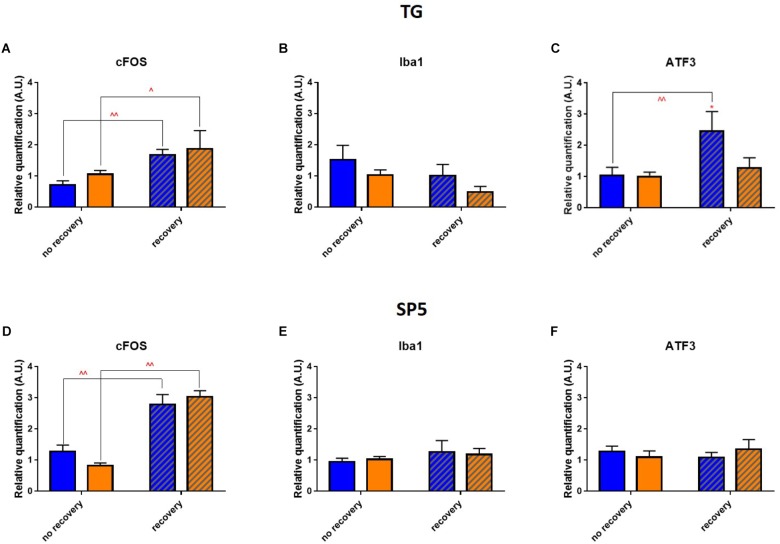
Phototoxicity marks in the trigeminal pathway. **(A–C)** Results of RT-qPCR analysis on the TGs: mRNA expression of cFOS **(A)**, Iba1 **(B)**, and ATF3 **(C)**. Statistical significance: cFOS: *blue* no recovery vs. recovery – *q* = 0.0129, *p* = 0.0061; *yellow* no recovery vs. recovery – *q* = 0.0339, *p* = 0.0323; ATF3: *blue* no recovery vs. recovery – *q* = 0.0076, *p* = 0.0072; *recovery* blue vs. yellow – *q* = 0.0243, *p* = 0.0231. **(D–F)** Results of RT-qPCR analysis on the brainstems: mRNA expression of cFOS (**D**; blue no recovery vs. recovery – *q* < 0.0001, *p* < 0.0001; yellow no recovery vs. recovery – *q* < 0.0001, *p* < 0.0001), Iba1 **(E)** and ATF3 **(F)**. Blue and yellow bars correspond to blue and yellow exposures, respectively; clear and hatched bars correspond to the time points of mice dissection, either directly after illumination (*no recovery*) or in 3 days of recovery (*recovery*), respectively. All the data are presented as mean ± SEM. Differences were considered significant when *p* < 0.05 (^∗^/^∧^), *p* < 0.01 (^∗∗^/^∧∧^), *p* < 0.001 (^∗∗∗^/^∧∧∧^) or *p* < 0.0001 (^∗∗∗∗^/^∧∧∧∧^). Stars correspond to comparisons between blue-illuminated and yellow-illuminated mice, treated with the same drug. Carets correspond to comparison of mice assessed directly after illumination to the ones after 3 days of recovery, within the same spectra. Red color means increase and blue color decrease in values.

To delineate the neuro-inflammatory circuit underlying the phototoxicity, we checked whether any inflammation was induced in trigeminal pathways. In the trigeminal ganglia (TGs) for both illuminations after 3 days of recovery, analysis of mRNA expression revealed a significant increase in cFOS rate (Figure 10A). In blue-illuminated mice after recovery, we detected an increase in ATF3 rate (Figure 10C). Moreover, blue light provoked a non-significant trend for Iba1 level increase immediately after the end of illumination (Figure 10B). We then studied whether the inflammation observed in the TGs was transmitted to the spinal trigeminal nucleus or *sp5* (Vi/Vc and Vc/C1 transition regions). Here, the RT-qPCR analysis revealed the same cFOS-pattern as the one observed in the TGs (Figure 10D). mRNA expression of Iba1 and ATF3 did not exhibit any significant difference (Figure 10E,F).

**FIGURE 11:**
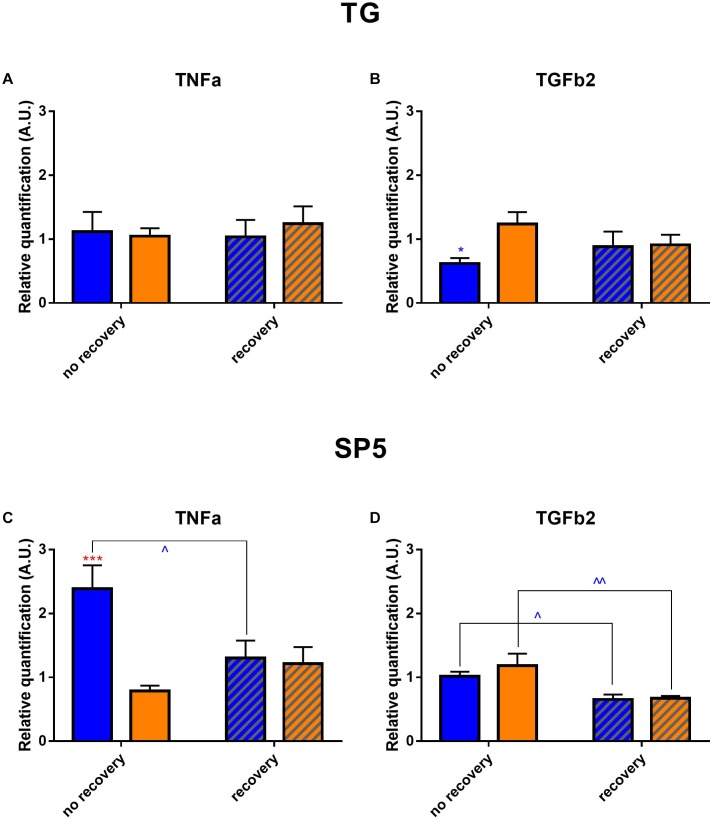
Cytokine profile in trigeminal pathways. Results of mRNA expression of TGFβ2 and TNFα on the TGs **(A,B)** and brainstems **(C,D)**. Statistical significance for the TG: TGFβ2 blue no recovery vs. recovery – *q* = 0.0187, *p* = 0.0178. Statistical significance for the brainstem; TGFβ2: blue no recovery vs. recovery – *q* = 0.0169, *p* = 0.0161; yellow no recovery vs. recovery – *q* = 0.0041, *p* = 0.0020; TNFα: blue no recovery vs. recovery – *q* = 0.0197, *p* = 0.0188; recovery blue vs. yellow – *q* = 0.0005, *p* = 0.0005. Blue and yellow bars correspond to blue and yellow exposures, respectively; clear and hatched bars correspond to the time points of dissection, either directly after illumination (no recovery) or in 3 days of recovery (recovery), respectively. All the data are presented as mean ± SEM. Differences were considered significant when *p* < 0.05 (^∗^/^∧^), *p* < 0.01 (^∗∗^/^∧∧^), *p* < 0.001 (^∗∗∗^/^∧∧∧^) or *p* < 0.0001 (^∗∗∗∗^/^∧∧∧∧^). Stars correspond to comparisons between blue-illuminated and yellow-illuminated mice, treated with the same drug. Carets correspond to comparison of mice assessed directly after illumination to the ones assessed after 3 days of recovery, within the same spectra. Red color means increase and blue color decrease in values.

Finally, we verified by RT-qPCR whether the phototoxicity induced an over-expression of TGFβ2 and TNFα since both cytokines are known to be highly involved in inflammation (Figure 11). In TGs directly after illumination, TGFβ2 rate was significantly decreased in blue-light samples as compared to the yellow-light ones. In brainstems for both light conditions, TGFβ2 expression went down after the recovery time when compared to its after-exposure level. In TGs, we did not detect any significant changes in TNFα rate; however, in brainstems, its level was importantly increased directly after blue-light exposure, but then went down after the recovery period.

## Discussion

Photophobia and specific hypersensitivity to blue light are common symptoms of many ocular diseases, foremost among them the dry eye. This issue has been gaining more attention since the spectra of modern light sources contain an important blue part. Nonetheless, the correspondent underlying mechanisms are still debated (Digre and Brennan, 2012; Matynia and Gorin, 2013; Marshall, 2014; Katz and Digre, 2016; Lupis, 2017; Text Request, 2017; Wu and Hallett, 2017; Albilali and Dilli, 2018). Here, we investigated *in vivo* the origins and effects of spectrum-dependent photophobia by means of behavioral and pharmacological studies in mice exposed to blue or yellow light.

### Blue-Light Aversion Is Accompanied by Clinical Signs of Dry Eye

Three-hour-exposure provoked stable light aversion in blue- but not in yellow-illuminated mice thus proving that this photophobic effect was wavelength-dependent and was not simply induced by bright light of random spectrum. As expected, the fluorescein staining test using slit-lamp examination did not reveal any epithelial damage in the cornea. Indeed, even if the average irradiance that we used (6 mW/cm^2^) was strong enough to induce light-aversive behavior, it was still within the range of irradiances one may get from daily sun exposure which is not supposed to noticeably injure the ocular surface. In comparison, to induce a significant increase in corneal fluorescent staining, Lee et *al*. exposed mice to blue light of 29.2 mW/cm^2^ irradiance with the entire radiant exposure of 1,000 J/cm^2^ while in our experiments it was 64.8 J/cm^2^ (Lee et al., 2016). The absence of outward signs cannot guarantee the absence of more intrinsic damage though. IVCM imaging revealed the inflammatory signs in epithelium, sub-basal plexus and stroma of mice exposed to blue light. We found that phototoxically induced inflammation accumulated in the cornea after 3 days of recovery, in line with Feng’s study (Feng et al., 2017). Exposure to yellow light did not provide with any important clinical signs of damage: IVCM images of yellow-light-illuminated corneas did not exhibit any significant difference with the naïve mice (Supplementary Figure S2). Since we were seeking to investigate the blue-spectrum-specific photophobia, we used the yellow illumination as the control lighting condition for our further experiments.

It has already been reported that physical disruption of the corneal surface and increased corneal nociception correlated with increased light aversion (Matynia et al., 2015). Here, we showed that exposure to blue light in itself provided with an increase in corneal mechanical sensitivity. In line with the IVCM data, this result worsened after the recovery time. In addition, blue light provoked an excessive tearing that might be ascribed to extra-blinking, induced by photophobia, that in turn provided with greater lacrimation. Indeed, this hypothesis, initially proposed by Digre and Brennan (2012), was then confirmed by Lei et al. (2018a) who reported an increased lacrimation in healthy humans exposed to blue light of 470 nm as compared to their baseline values. Taken together, these data demonstrate clinically that blue-light aversion is accompanied by increased inflammation within the cornea as well as by altered lacrimation reflex. Since these clinical signs are the ones frequently observed in dry eye patients (Belmonte et al., 2017; Bron et al., 2017; The National Eye Institute, 2017), our study confirms that blue-light exposure may provoke and/or aggravate the dry eye disease, as it has been supposed previously (Niwano et al., 2014; Ayaki et al., 2015; Lee et al., 2016; Marek et al., 2018). Alterations in lacrimation reflex might be also ascribed to the decreased lacrimal gland innervation observed in dry eye by Chen et al. (2017); this issue is all the more important given that we recently reported the phototoxicity in trigeminal innervation provoked by blue-light exposure (Marek et al., 2019).

#### Retinal Mediation in the Blue-Toxic Process

Retina is the most well-known center for photic signal reception, processing and transmission to the brain. Numerous studies demonstrated the phototoxic impact of bright blue light on various retinal structures, *in vivo* (Jaadane et al., 2015; Krigel et al., 2016; Lee et al., 2016; Feng et al., 2017), *ex vivo* (Roehlecke et al., 2013), and *in vitro* (Godley et al., 2005; Lascaratos et al., 2007; Arnault et al., 2013; Marie et al., 2013). That is why we first checked whether our light protocol, less aggressive than the one usually reported and therefore closer to daily light conditions, induced any damage in the retina. Expectedly, by means of immunochemistry and of RT-qPCR, we detected increased activation of Müller cells (anti-GFAP staining) and microglia (anti-Iba1 staining). Phototoxically induced inflammation accumulated during 3 days thus resulting in more important signal after the recovery time, in line with previous reports (Feng et al., 2017). The GFAP-stain in our experiments was less pronounced than in the work of Krigel et al. (2016) and Feng et al. (2017) since in our illumination protocol, the irradiance and exposure time were much less important. One should note that microglial activation was also detected after exposure to yellow light, probably due to greater illuminance of yellow light as compared to the blue one in terms of photometric units (i.e., in lux, as perceived by the human eye). In addition, in RGCs, we observed the activation of ATF3, a mediator of cellular stress response and a regulator of cellular proliferation. ATF3 is either not expressed or expressed at very low levels in most intact neurons *in vivo* (Hunt et al., 2012). Since it is an immediate early stress-inducible gene, we expectedly detected it directly after the end of light exposure. Moreover, it has already been reported that light provided with damage in retinal photoreceptors (Contín et al., 2013; Jaadane et al., 2015; Krigel et al., 2016). Indeed, we observed a morphological degradation of the cone layer in retinas of mice exposed to blue but not to yellow light. Taken together, these results confirm that large-spectrum blue light, even of smaller radiant exposure, does provoke retinal inflammation and visual receptors damage.

We then tried to modulate the photophobic behavior by altering the luminous flux that entered the eye. Expectedly, pupil dilatation (atropine instillation) provoked the aversion to yellow light that did not take place previously, in line with the results of Matynia et al. (2012). Indeed, starting from a certain threshold, light of any spectrum naturally becomes dazzling. As for the time spent under the blue light, atropine instillation did not decrease it significantly since the smaller flux of light (without atropine) was already sufficient to completely turn mice away from light.

Next, we supposed that light aversion might be overcome by disruption of pathways used by retinal visual receptors. Even if mice injected with the correspondent drug (VR blocker) were blind (Supplementary Figure S1), the induced absence of image-forming vision did not provide with any significant impact on light-aversive behavior. This result is corroborated by the fact that blind patients (Digre and Brennan, 2012; Albilali and Dilli, 2018) as well as mice with ablated rods and cone photoreceptors (Matynia et al., 2012) can still exhibit the photophobic symptoms. Thus, we concluded that the role of image-forming vision (and therefore of visual photopigments) in mediation of spectrum dependent photophobia is not the major one.

We then investigated whether the non-visual light receptors might be responsible for the photophobic behavior. The most well-known light-sensitive and non-visual retinal pigment is melanopsin (opn4). It is present in 2–3% of RGCs (called ipRGC) that regulate circadian rhythms, PLR and other behavioral and physiological responses to environmental illumination (Berson, 2003). Importance of ipRGC in bright light aversion has been extensively reported by Matynia et al. (2012, 2015). In our study, we found that mRNA level of melanopsin was decreased directly after the light exposure when compared to its level after the recovery time. This result is in line with those of Hannibal et al. (2005) who reported the decrease in melanopsin mRNA level during exposure to constant light. They also found that illumination decreased melanopsin immunostaining in a time-dependent manner, starting from the distal dendrites and going to the proximal dendrites and the soma. We observed the dotted structure in the dendrites of retinas dissected directly after illumination and the disappearance of anti-melanopsin stain in distal dendrites after the recovery period. This follows Benedetto et al. (2017) who exposed rats to constant light for 2–8 days and observed decreased levels of melanopsin retinal immunoreactivity in distal neurites.

Benedetto et al. (2017) also reported the increased levels of anti-neuropsin (anti-opn5) immunolabeling in some cells of GCL and INL. This non-visual photoreceptor is gaining today an increasing attention. Its presence and importance for photoentrainment have been discovered in the retina and cornea; however, its precise functions are still not clear (Guido et al., 2011; Buhr et al., 2015). Nonetheless, role of opn5 in photophobia management might be suspected from the results some recent studies (Hughes et al., 2016; Matynia et al., 2017). We therefore checked the status of neuropsin by immunochemistry and RT-qPCR but did not find any significant differences between the two spectra. This discrepancy could be due to the fact that Benedetto et al. (2017) observed increasing levels of neuropsin after 4 days of exposure to light while we illuminated mice only during 3 h. In addition, the illumination protocol we used was much different from their one (in terms of light spectrum, irradiance and exposure time).

Thus, we hypothesized that melanopsin might be the main blue-light mediator for photophobia. We performed a behavioral test with mice injected with melanopsin antagonist reported to specifically modify melanopsin-dependent light responses (Jones et al., 2013; Sikka et al., 2014). Indeed, such injection did significantly reduce the blue-light aversion. It did not provoke the yellow-light aversion like atropine instillation since this antagonist does not dilate the pupil in longer term (Supplementary Figure S3); the action of antagonist on the PLR is shorter than on the ipRGC activity itself (Jones et al., 2013). Thus, it did not alter significantly the ipRGC-independent behavior of mice under yellow light while reducing the ipRGC-dependent blue-light aversion.

**FIGURE 12:**
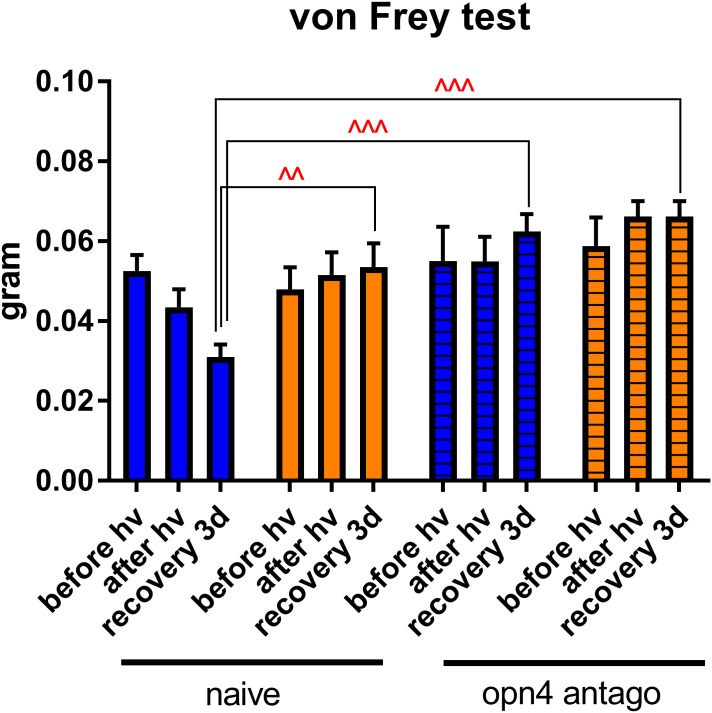
Role of melanopsin in corneal sensitivity. Measurement of corneal mechanical sensitivity performed by means of von Frey test. Greater values mean lower corneal sensitivity. Test was performed in naïve mice (clear bars, the same results as the ones presented in the Figure 2) and in mice intraperitoneally (*ip*, 30 mg/kg) injected with melanopsin antagonist (*opn4 antago*) 15 min before the start of the test. For more detail, see “Materials and Methods” section. Statistical significance for the *recovery 3d* grou*p*: blue naïve vs. yellow naïve – *q* = 0.0010, *p* = 0.0010, blue naïve vs. blue antago opn4 – *q* = 0.0008, *p* = 0.0005, blue naïve vs. yellow antago opn4 – *q* = 0.0004, *p* = 0.0001. Measurements were made at three time points: *before hν*, before the beginning of illumination; *after hν*, directly after 3 h of illumination; *recovery 3d*, after 3 days of recovery in standard illumination conditions of animal unit. Blue and yellow bars correspond to blue and yellow exposures, respectively. All the data are presented as mean ± SEM. Differences were considered significant when *p* < 0.05 (^∧^), *p* < 0.01 (^∧∧^), *p* < 0.001 (^∧∧∧^) or *p* < 0.0001 (^∧∧∧∧^).

In addition, we measured corneal mechanical sensitivity (von Frey hair test) in mice that were injected with melanopsin antagonist before light exposure. Strikingly, we found that in these mice, corneal sensitivity did not increase as it did in the naïve ones (Figure 12). This result is in compliance with the study of Matynia et al. (2015); again, it highlights the crucial role of melanopsin in corneal nociception.

### Implication of Out-Retinal Melanopsin and Activation of Trigeminal Pathways

The retina is not the only mammalian tissue that contains melanopsin. This photopigment was also found in iris (Xue et al., 2012), ciliary body (Semo et al., 2014), and blood vessels (Sikka et al., 2014). The team of Matynia discovered that melanopsin was expressed in 3% of small TG neurons localized in the ophthalmic branch of the trigeminal nerve, and reported their intrinsic photosensitivity, as well as showed the presence of melanopsin transcripts in the cornea (Matynia et al., 2016). Very recently, Delwig et al. (2018) discovered the previously unrecognized localization of melanopsin protein in corneal nerve fibers using antibody staining. In our preliminary immunochemistry and PCR electrophoresis experiments, we had detected the presence of melanopsin in the cornea, TGs and brainstem (data not shown).

We were then wondering whether the non-retinal melanopsin-containing tissues might have a role in mediation of blue light photophobia. We performed a behavioral test to assess light aversion in mice instilled with oxybuprocaine. This topical anesthetic numbed the entire surface of the eye thus disrupting the nociceptive transmission from all the ocular surface neurons among which are melanopsin-containing ones (Supplementary Figure S4). We did not observe any difference in oxybuprocaine-instilled mice as compared to the PBS-instilled ones. According to our clinical practice, oxybuprocaine has a peak in action in 1–15 min after the instillation; the correspondent anesthetic effect lasts till 45 min. To make sure that we did not miss any short-term effect that oxybuprocaine might have provided with, we checked the results of behavioral test at various time points within the first hour; however, we still did not observe any important difference (Supplementary Figure S5A). This result is in accordance with those of Lei et al. (2018b) who found that topical ocular anesthesia did not alter the psychophysical photophobia thresholds for either blue or red light in humans. Moreover, Delwig et al. (2018) reported the absence of light responses in the melanopsin-expressing corneal fibers. Thus, we may conclude that nerve fibers within the cornea make little contribution, if any, to photophobia.

Next, we investigated the role of photophobic pathways proposed by Okamoto et al. (2009, 2011, 2012) and Rahman et al. (2015). According to the authors, light signal, firstly received and processed by the retina, could then activate intraocular TG nerves. This might happen either by transmitters released from parasympathetic postganglionic neurons or, for those fibers apposed to blood vessels, by mechanical deformation of the latter due to changes in blood flow. To check these hypotheses, we injected mice intravitreally either with lidocaine, which blocks the nociceptive trigeminal near-retinal afferents present within the eye, or norepinephrine that constricts potentially dilated blood vessels. None of these pharmacological treatments provided with a significant change in behavior under blue light. According to Okamoto et al. (2009, 2011, 2012), the effect of lidocaine and norepinephrine disappeared in 40–50 min after injection, so we checked the behavior at shorter time periods. We still did not observe anything significant (Supplementary Figures S5B,C), in disagreement with this group who reported the complete block of light-evoked neural activity. Nevertheless, in our results, one should note the trend for blue-light aversion decrease. Again, we might put down this discrepancy to important differences in experimental protocols, either in illumination (they used 30 s white light stimuli of 10^4^ lux) or in light impact assessment (electrophysiology). Surprisingly, in our study, lidocaine injection induced yellow light aversion. According to our clinical practice, anesthetic intravitreal lidocaine injection may make patients slightly blind. Indeed, lidocaine blocks the voltage-gated Na channels; in addition to the nociceptive afferents, these channels are present in amacrine cells that participate in the integration of visual signals the retina (Tian et al., 2010). By measuring the optokinetic response, we verified that in mice, lidocaine ivt injection induced significant blindness as compared to control or norepinephrine ivt injection (data not shown). We therefore supposed that lidocaine-injected mice were not able to detect yellow light anymore, thus spending approximately half of illumination time in the dark and another half in the light part of the box.

Okamoto et al. (2009) also observed light-induced neuronal activation in trigeminal brainstem of rats. In humans during photophobia periods, Moulton et al. (2009) reported fMRI-detected specific activation patterns at the level of the TG, trigeminal nucleus caudalis, and ventroposteromedial thalamus. In our team, we already reported an activation of trigeminal pathways in response to corneal inflammation (Launay et al., 2016). Accordingly, in the current study, we observed an activation in the TGs and brainstems.

### Probable Pathways of Blue Light Photophobia

**FIGURE 13:**
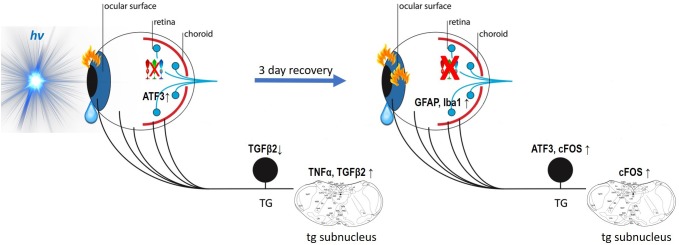
Model of blue-phototoxicity. Proposed scheme of blue light provoked time-dependent immune and inflammatory responses in the ocular surface, trigeminal pathways and the retina. For more detail, see the “Discussion” section.

To our knowledge, it is the first *in vivo* study to report the spectral selectivity of photophobia in light conditions close to that of the daily living (inferred from behavior assessment supplemented by various pharmacological treatments). We demonstrate that blue-light exposure provokes important immune and inflammatory responses in the ocular surface, trigeminal pathways and the retina. In dependence on the time of assessment and on the tissue, these responses were induced and spread differently. Our results might be integrated in a model of blue-phototoxicity as follows (Figure 13).

In the ocular surface, the immediate answer to the 3 h of blue light illumination is the immune response of dendritic cells and activated keratocytes. In the trigeminal pathway, it reveals itself in alterations in cytokines’ (TGFβ2, TNFα) gene expression that during the following 3 days of recovery, cause neural activation and inflammation (ATF3, cFOS). Moreover, immunoreactions in the trigeminal and ocular surface structures [in which the blue light phototoxicity has been recently reported by our team (Marek et al., 2019)] provoke an increase in corneal mechanical sensitivity and extra-lacrimation.

In the retina, the events sequence of the phototoxic reaction is quite the opposite. The immediate answer to the blue-light exposure is revealed by a small loss of cone photoreceptors and an increase in the immediate early gene response to stress (ATF3). During the recovery, cone apoptosis becomes more important, therefore attracting macrophages (Iba1) and activating Müller cells (GFAP). The reasons for various orders in phototoxic processing need further investigation.

We show the crucial role of melanopsin in corneal mechanical sensitivity and report that blue light photophobia is mainly mediated by melanopsin-containing cells and does not rely on visual receptors. Despite that, the intra-corneal melanopsin-containing trigeminal fibers seem to have a minimal role in intrinsic photosensitivity. The fact that we, however, observed the trigeminal inflammation may mean that the photic signal is received by the ipRGC and then somehow transmitted to the trigeminal pathways, simultaneously inducing the phototoxic stress in the retina. According to our results, this process doesn’t implicate the blood flow alterations. Phototoxic message transfer might happen by means of light-induced transmitters released to intraocular TG afferents at the posterior part of the eye; however, this pathway did not appear to be the main one.

There are other possibilities to transmit the phototoxic message from the retina to the trigeminal system. First, the group of Matynia proposed the ipRGC-independent alternative pathway of light avoidance that was unmasked by morphine sensitization (Matynia et al., 2012, 2017); however, the precise operation of this circuit remains to be clarified. Second, Dolgonos et al. (2011) suggested an intra-retinal processes, independent on central visual centers, that could produce an enhanced trigeminal response to light. They proposed that so-called *associational retinal ganglion cells*, that did not enter the optic nerve, extended into the retinal periphery near the ciliary body. Since this region is richly innervated with trigeminal nociceptors, associational RGC might directly sensitize the neurons of sp5. Third, we cannot exclude the probable roles of iris and ciliary body. Iris was reported to contain melanopsin (Xue et al., 2012); since it is innervated by the trigeminal sensory fibers (McDougal and Gamlin, 2015), iris would be able to receive and transmit the photic signal in the trigeminal system. Semo et al. (2014) identified melanopsin-rich CMZ plexus and observed melanopsin-positive fibers projecting from ipRGCs at the CMZ directly into the ciliary body. They also reported that melanopsin was expressed at low levels in the ciliary body itself (Semo et al., 2014). In our experiments, these last two pathways were not affected by lidocaine injection since its numbing action might be strongly attenuated while spreading through the vitreous body and the choroid. In addition, although it was initially believed that the retina lacked trigeminal sensory innervation (Albilali and Dilli, 2018), recently Warfvinge et al. (2018) reported that nerves originating from the TGs did innervate the retina. Moreover, this group has already detected the presence of CGRP neuropeptide in the rat retina (Blixt et al., 2017); this peptide was shown to colocalize with melanopsin in human post mortem trigeminal neurons by Matynia et al. (2016). In our experiments, we also observed anti-CGRP immunostaining in GCL and INL (Supplementary Figure S6). These findings support the possibility of direct communication between the TGs and ipRGC. Further investigations of retinal and near-retinal connection to the trigeminal system would allow for better understanding of blue-light aversive behavior mechanisms and consequently for better and targeted treatment for photophobic patients.

## Materials and Methods

### Animals

Adult male C57BL/6 mice (30 g; Janvier Labs, Le Genest Saint Isle, France) were maintained under controlled conditions (22 ± 1°C, 60 ± 10% relative humidity, 12/12 h light/dark cycle, food and water *ad libitum*). All experiments were approved by the Charles Darwin Ethics Committee for Animal Experimentation (Ce5/2011/05) and carried out in accordance with Directive 2010/63/EU of the European Parliament and the Council of 22 September, 2010 and French law (2013/118).

Before the beginning of all the experiments, mice spent 1 week in standard conditions of animal facility; during this period, they were daily handled to be habituated to the experimenter. Animals were weighted before treatment and at the end of the experiments.

### Light Protocol (Figure 1)

Mice were illuminated for 3 h by custom-mounted commercial LED sources (AVAB Transtechnik France, St. Denis, France) of blue and yellow spectra (Figure 1A); the corresponding characteristics are summarized in the Table 1. All the light exposures took place between 9 a.m. and 1 p.m. All the experiments were performed either directly after the end of light exposure or in 3 days of recovery in standard lighting conditions of animal facility. For the clinical assessments, RT-qPCR and immunohistochemistry, mice were placed in separate compartments of mirrored-wall cages. For the behavioral tests, mice were placed in separate half-illuminated boxes where they could move freely between illuminated and darkened parts (Figure 1C). For every experiment, cages and boxes were carefully cleaned. Mice were not able to observe each other or to interact.

**Table 1:** Spectral and intensity characteristics of customized light source.

	Waveband	Average irradiance	Radiant exposure	Average illuminance
Blue	400–500 nm	6 mW/cm^2^	64.8 J/cm^2^	400 lux
Yellow	530–710 nm	6 mW/cm^2^	64.8 J/cm^2^	3,500 lux


### Behavior Tests (Figure 1C)

To acclimatize to the experiment conditions, mice were placed in half-illuminated boxes 10 min before the start of exposure. Animals were filmed during all the time of illumination. For every hour, time spent in the illuminated part of the box was calculated for the following representative periods: 0–5, 20–25, 40–45, and 55–60 min; the values were then summed up.

### Pharmacology

Applied drugs are described in the Table 2. All the instillations and ivt injections were performed bilaterally. Instillation volume corresponded to 1 drop per eye (delivered by a micropipette). For ivt injections, the animal was anesthetized by means of isoflurane (5% then 2%), then the globe was pierced through the sclera posterior to the limbus by a 30 gauge needle, than the drug (2 μL/eye) was delivered from a 33 gauge needle.

**Table 2:** Name, manufacturer, method, and time of application (relative to the beginning of light exposure) and bibliographic reference (if applicable).

Drug	References	Time before exposure	Method of use	Literature
PBS	Life Technologies, Carlsbad, CA, United States	Directly before	Instillation or ivt injection	NA
Atropine sulfate 1%	Europhta, Monaco	5 min	Instillation	Okamoto et al., 2011; Matynia et al., 2012
Pilocarpine nitrate 1%	Europhta, Monaco	15 min	Instillation	NA
Visual receptors (VR) blocker	*See below*	5 min	ivt injection	Bush and Sieving, 1994; Gregory Gauvain, personal communication
opn4 antagonist 30 mg/kg	Merck, St Quentin en Yvelines, France	15 min	ip injection	Xue et al., 2012; Jones et al., 2013
DMSO HYBRI-MAX	Sigma-Aldrich, St. Louis, MO, United States	15 min	ip injection	NA
Oxybuprocaine hydrochloride 1.6 mg/0.4 mL	Thea, Clermont-Ferrand, France	Directly before	Instillation	NA
Lidocaine hydrochloride 2%	Aguettant, Lyon, France	5 min	ivt injection	Okamoto et al., 2011
DL-norepinephrine hydrochloride 10 mM (Supplementry Figure S7)	Sigma-Aldrich, St. Louis, MO, United States	5 min	ivt injection	Okamoto et al., 2011


*NA, not applicable.*

To prepare the “visual receptor blocker” (*VR blocker*) drug, 40 mM of L-AP4 (Tocrys, Biotechne, Lille, France) was mixed with 200 mM of PDA (Abcam, Paris, France). L-AP4 (L-(+)-2-amino-4-phosphonobutyric acid) is a glutamate receptor agonist and therefore blocks synaptic transmission at the synapse between photoreceptors and ON bipolar cells. PDA (2,3 *cis*-piperidine dicarboxylic acid) is an ionotropic receptor antagonist; it suppresses transmission at the synapse between photoreceptors and OFF bipolar cells and horizontal cells (Bush and Sieving, 1994). For each eye, 0.25 μL of this solution was added to 1.75 μL of PBS.

Opn4 antagonist was diluted in DMSO 100%, as proposed by the supplier; applied concentration was 30 mg/kg of dry antagonist that resulted in 50–60 μL of diluted antagonist per animal approximately.

### Clinical Assessment

The following clinical assessments were implemented one after another either directly after the end of illumination or after 3 days of recovery in standard lighting conditions.

• Corneal mechanical sensitivity (von Frey hair test)

Mechanical stimulation was performed with calibrated von Frey hairs of increasing force (0.008–0.07 g) applied for 1 s to the cornea (de Castro et al., 1998). The response to the stimuli was determined as positive when the mouse presented a complete blink.

• Tear volume (phenol red test)

Tear production was measured with the phenol red thread test (Zone-Quick; Lacrimedics, Eastsound, WA, United States). The threads were placed in the lateral canthus of the conjunctival fornix of the eye for 30 s as previously described (Launay et al., 2016). The thread is initially yellow in color (acidic); when exposed to tears, it changes its color to a red one. After 30 s, the “tear distance” (in millimeters) was determined using a provided scale.

• *In vivo* confocal microscopy (IVCM)

A laser-scanning *in vivo* confocal microscope [IVCM, Heidelberg Retina Tomography (HRT)] with II/Rostock CorneaModule (RCM) (Heidelberg Engineering, GmbH, Heidelberg, Germany) was used to examine the entire cornea of anesthetized mice [by ip injection of 150 μL mixture of Ketamine 1000 U (100 mg/kg body weight) and xylazine (10 mg/kg bodyweight) (Virbac, France)] as described previously (Launay et al., 2016). Shown images illustrate the representative state of corneal layers for all the animals.

### RT-qPCR

Mice were deeply anesthetized with 200 μL mixture of ketamine 1000 U (100 mg/kg body weight) and xylazine (10 mg/kg bodyweight) (Virbac, France) injected intraperitoneally. Animals were then perfused with cold (4°C) 10 mL 0.9% NaCl solution and the retinas, TGs and brainstems were carefully dissected and placed immediately in liquid nitrogen until the extraction procedure.

RNAs were extracted from TGs, retinas, and brainstems using the Macherey-Nagel NucleoSpin RNA extraction kit, according to the manufacturer’s protocol. (Macherey-Nagel, Düren, Germany). RNA quality and quantity were assessed using a ND-1000 spectrophotometer (Thermo Scientific, Waltham, MA, United States). cDNA was further synthesized from equal amounts of RNA using Multiscribe reverse transcriptase (TaqMan Reverse Transcription Reagents, Applied Biosystems, Life Technologies, Carlsbad, CA, United States) according to the manufacturer’s protocol. Finally, cDNA were diluted in DNAse/RNAse free water (Gibco) to a final concentration of 5 ng/μL. Real-time quantitative PCR was performed with 25 ng of cDNA added to a 15 μL solution of Applied Biosystems Mastermix (TaqMan Universal PCR Master Mix) and primers to a final volume of 20 μL. All primers and reagents were purchased from Applied Biosystems: GAPDH (Mm99999915.m1), ATF3 (Mm00476032.m1), FOS (Mm00487425.m1), GFAP (Mm01253033.m1), Iba1 (Mm00479862.g1), opn4 (Mm00443523.m1), opn5 (Mm00710998.m1), TNF-α (Mm99999068.m1), and TGFβ2 (Mm00436955.m1). Target cDNA was amplified using the 7300 Real-Time PCR system (Applied Biosystems). Changes in mRNA expression were calculated as ΔΔCt = ΔCt_illuminated_ – ΔCt_control_ with ΔCt = Ct_target_gene_ – Ct_HK_gene_. Ct means *cycle threshold* and HK_gene means *housekeeping gene* (GAPDH). Tissues of yellow-illuminated mice dissected directly after light exposure were taken as controls. Since our aim was to investigate the spectral characteristics of photophobia and especially its blue specificity, normalization to the gene expression rates of naïve non-illuminated mice would not provide us with the information relevant to the scope of this study.

### Immunochemistry

Mice were deeply anesthetized with 200 μL mixture of ketamine 1000 U (100 mg/kg body weight) and xylazine (10 mg/kg bodyweight) (Virbac, France) injected intraperitoneally. Animals were then perfused via the ascending aorta with 5 mL of 0.9% NaCl solution followed by 30 mL of 4% paraformaldehyde solution. After fixation, eyes were carefully dissected out and post-fixed 48 h in the same fixative. Retinas were dissected from the eyes and placed sequentially in 10, 20, and 30% sucrose solution in 1× PBS, overnight for each treatment, immerged in OCT (Tissue-Tek^®^ O.C.T. Compound, Sakura^®^ Finetek) and finally frozen in liquid nitrogen. Cryostat sections (Leica, Germany) of 12 μm were then performed and mounted on Superfrost slides; sections were kept at -20°C until use.

After three washes in 1× PBS, tissues were placed in a blocking buffer (3% normal donkey serum, 0.3% triton) for 2 h, then incubated at 4°C for 24 h with the following primary antibodies diluted in blocking buffer: rabbit anti-ATF3 (Santa Cruz Biotechnology, sc-188, 1/500), chicken anti-GFAP (Thermo Fisher Scientific, PA1-10004, 1/1000), rabbit anti-Iba1 (Wako, 019-19742, 1/500), rabbit anti-opn4 (ATS, AB-N39, 1/500), rabbit anti-opn5 (Biorbyt, orb223499, 1/500), rabbit anti-Cone Arrestin (Merck, AB15282, 1/10000). For visualization, cells were incubated with the corresponded Alexa Fluor secondary antibodies (1:500 in PBS, Invitrogen) for 1 h at RT.

For all the immunostainings, negative control experiments (without incubation with a primary antibody) were performed, in order to ensure the absence of non-specific fluorescent signal. DAPI coloration is not presented to allow for better visualization of immunostaining of interest.

### Imaging

Samples were imaged with the microscope AXIO Imager.M1 (Zeiss, Germany). Images were recorded via provided ZEN software and then processed with the Fiji (ImageJ version). Identical exposure settings, that minimized oversaturated pixels in the final images, were used for both illumination and recovery (or not) conditions.

### Statistical Analysis

All the experiments were performed on minimum eight animals in every group. Statistical analysis was done using GraphPad (GraphPad Software, La Jolla, CA, United States). Two-way ANOVA analysis with repeated or non-repeated measures followed by false discovery rate multiple correction (two-stage step-up method of Benjamini, Krieger, and Yekutieli, false discovery rate *Q* = 0.05) were used. All the data are presented as mean ± SEM. Differences were considered significant when *p* < 0.05 (^∗^/^∧^), *p* < 0.01 (^∗∗^/^∧∧^), *p* < 0.001 (^∗∗∗^/^∧∧∧^) or *p* < 0.0001 (^∗∗∗∗^/^∧∧∧∧^). ^∧^! sign means that the difference was significant according to GraphPad software, although the *p*-value was slightly above 0.05. Blue and yellow bars correspond to blue and yellow exposures, respectively. Red color means increase and blue color decrease in values.

## Ethics Statement

All experiments were approved by the Charles Darwin Ethics Committee for Animal Experimentation (Ce5/2011/05) and carried out in accordance with Directive 2010/63/EU of the European Parliament and the Council of 22 September, 2010 and French law (2013/118).

## Author Contributions

VM and SMP conceived and designed the work. VM performed the experiments, analyzed and interpreted the results of experiments, and wrote the manuscript. ER, JD-C, AC, AD-L, AR-LG, and SMP were significantly involved in experiments realization and data analysis. TV, AD, and CB were involved in the design of the study. AR-LG and SMP reviewed the manuscript.

## Conflict of Interest Statement

VM and TV are employed by company Essilor International. The remaining authors declare that the research was conducted in the absence of any commercial or financial relationships that could be construed as a potential conflict of interest.

## Supplementary Material

FIGURE S1 Optokinetic test results. Optokinetic test proving the effect of the VR blocker ivt injection. Indeed, mice injected with this cocktail (white bars) demonstrated significantly reduced optokinetic answer as compared to PBS injection (gray bars), either in one or in three hours after the treatment. All the data are presented as mean ± SEM.

FIGURE S2 IVCM results of naïve mice. Representative images of non-invasive IVCM examination performed in mice kept under standard lighting conditions of animal facility. No significant difference with yellow-illuminated mice (Figure 3) was observed. The three following corneal layers are represented: superficial epithelium, sub-basal plexus (dendritic cells are marked by circles) and stroma (activated keratocytes are marked by arrows). In these mice, corneal mechanical sensitivity (von Frey hair test) was 0.043 ± 0.03 g.

FIGURE S3 Opn4 antagonist action on the PLR. Measurements of the pupillary reflex at various time points in mice ip injected with the melanopsin antagonist (gray bars) as well as in the control ones (black bars). All the data are presented as mean ± SEM. No significant changes were observed.

FIGURE S4 Corneal mechanical sensitivity after oxybuprocaine instillation. Von Frey test was realized at various time points after oxybuprocaine instillation (gray bars) and compared to the control condition (black bar). All the data are presented as mean ± SEM.

FIGURE S5 Shorter periods of behavioral tests. Graphs represent how the time spent in the illuminated part of the cage evolved during the 1st hour when oxibuprocaine **(A)**, lidocaine **(B)** or norepinephrine **(C)** were applied. Numbers 0–5, 20–25, 40–45, and 55–60 (min) correspond to time periods within the 1st hour. Blue and yellow bars correspond to blue and yellow exposures respectively; clear bars and hatched bars correspond to animals with control (vehicle – PBS) or specific drug treatments respectively. All the data are presented as mean ± SEM. Stars correspond to comparisons between blue-illuminated and yellow-illuminated mice, treated with the same drug. Carets correspond to comparisons between control and drug-treated animals. Red color means increase and blue color decrease in values.

FIGURE S6 Potential link between the retina and TG. Immunostaining of the retina with anti-CGRP antibody. On the merged image, CGRP- and DAPI-stainings are shown in green and blue respectively; spots of specific CGRP-staining are indicated by arrows. Magnification is 20x, scale bar corresponds to 100 μm.

FIGURE S7 Mydriatic action of norepinephrine. Measurements of the pupillary reflex at various time points in mice ivt injected with norepinephrine (gray bars) as well as in the control ones (black bars). All the data are presented as mean ± SEM.

## Abbreviations

antago: antagonist (on the behavioral tests graphs)
atro: atropine (on the behavioral tests graphs)
b – *blue*: blue-illuminated mice (on immunochemistry images)
CMZ: ciliary marginal zone
GCL: ganglion cell layer
INL: inner nuclear layer
ip: intraperitoneal
ipRGC: intrinsically photosensitive RGC
IVCM: *in vivo* confocal microscopy
ivt: intravitreal
lido: lidocaine (on the behavioral tests graphs)
norip: norepinephrine (on the behavioral tests graphs)
ONL: outer nuclear layer
oxybu: oxybuprocaine (on the behavioral tests graphs)
pilo: pilocarpine (on the behavioral tests graphs)
PLR: pupillary light reflex
nRec: *no recovery* – assessment directly after illumination (on the immunochemistry images)
Rec: *recovery* – assessment after 3 days of recovery (on the immunochemistry images)
RGCs: retinal ganglion cells
RPE: retinal pigment epithelium
sp5: spinal trigeminal nucleus
TG: trigeminal ganglion
VR: visual receptors
y – *yellow*: yellow-illuminated mice (on the immunochemistry images)
