# Supplementary figures and images for: Implication of Melanopsin and Trigeminal Neural Pathways in Blue Light Photosensitivity in vivo
Source: Front Neurosci. 2019 May 22;13:497. doi: 10.3389/fnins.2019.00497 (PMC6543920; doi:10.3389/fnins.2019.00497)

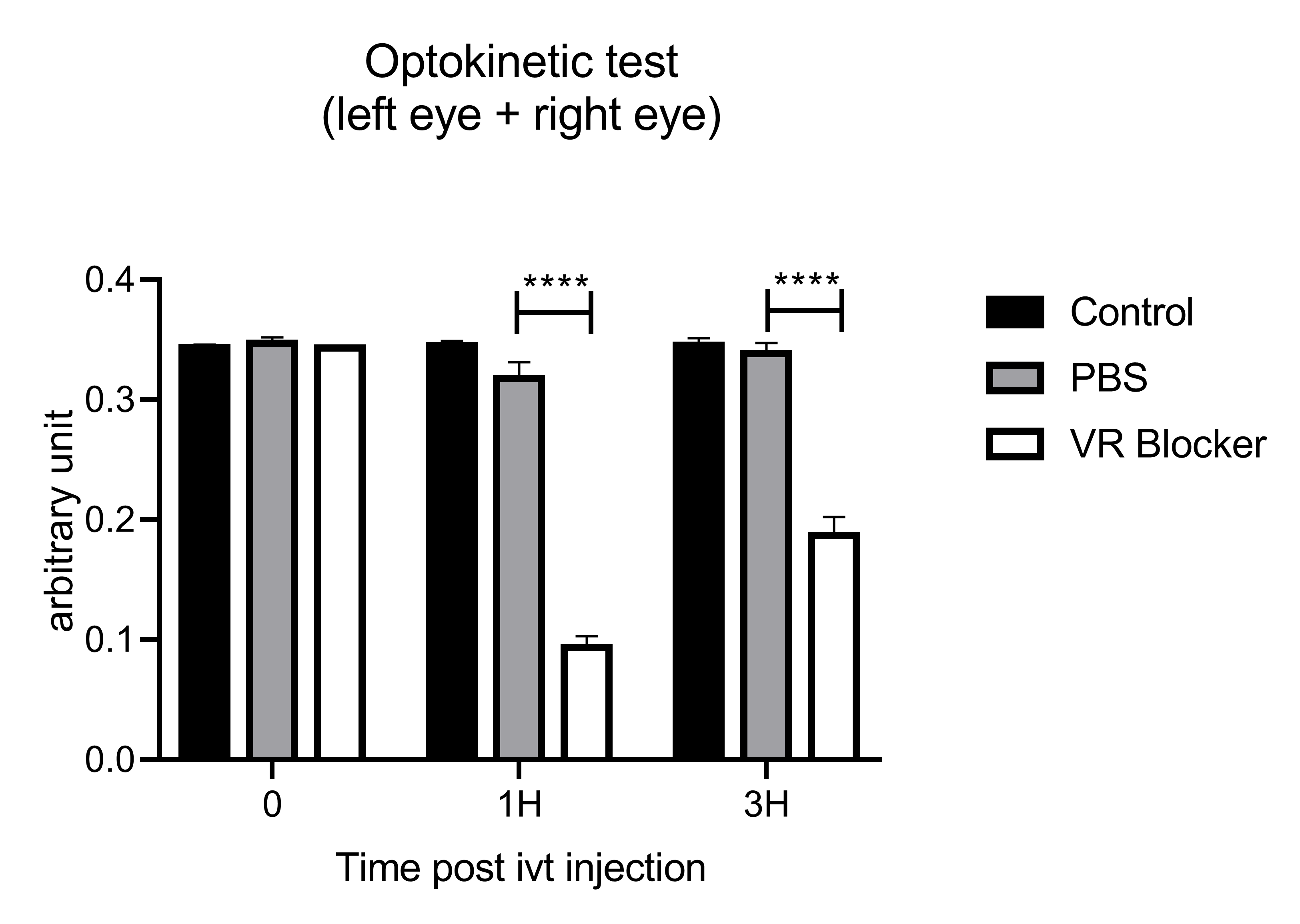

Supplement: FIGURE S1 — Optokinetic test results. Optokinetic test proving the effect of the VR blocker ivt injection. Indeed, mice injected with this cocktail (white bars) demonstrated significantly reduced optokinetic answer as compared to PBS injection (gray bars), either in one or in three hours after the treatment. All the data are presented as mean ± SEM. [file Image_1.TIF]

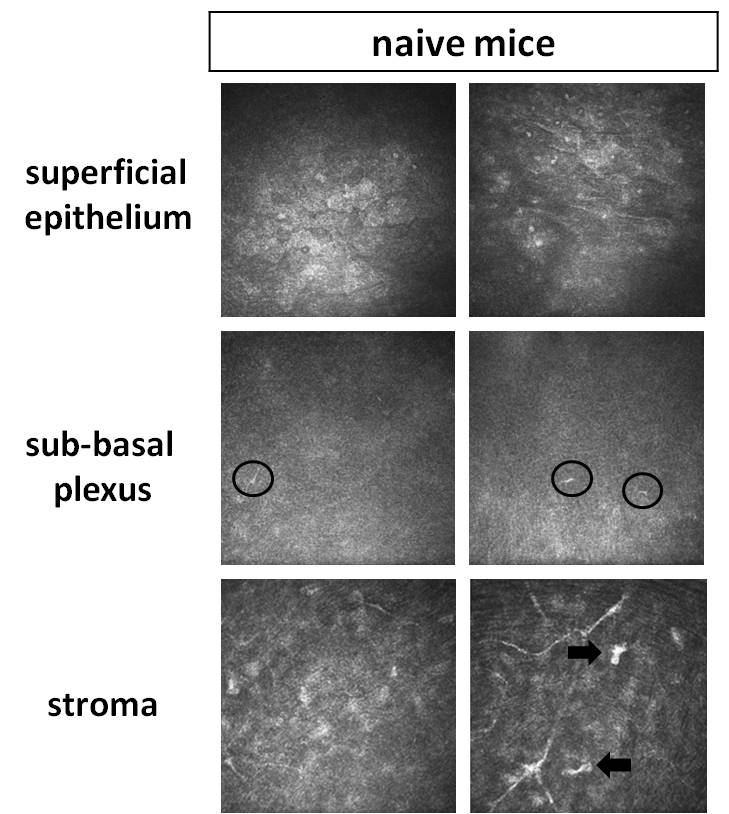

Supplement: FIGURE S2 — IVCM results of naïve mice. Representative images of non-invasive IVCM examination performed in mice kept under standard lighting conditions of animal facility. No significant difference with yellow-illuminated mice (Figure 3) was observed. The three following corneal layers are represented: superficial epithelium, sub-basal plexus (dendritic cells are marked by circles) and stroma (activated keratocytes are marked by arrows). In these mice, corneal mechanical sensitivity (von Frey hair test) was 0.043 ± 0.03 g. [file Image_2.JPEG]

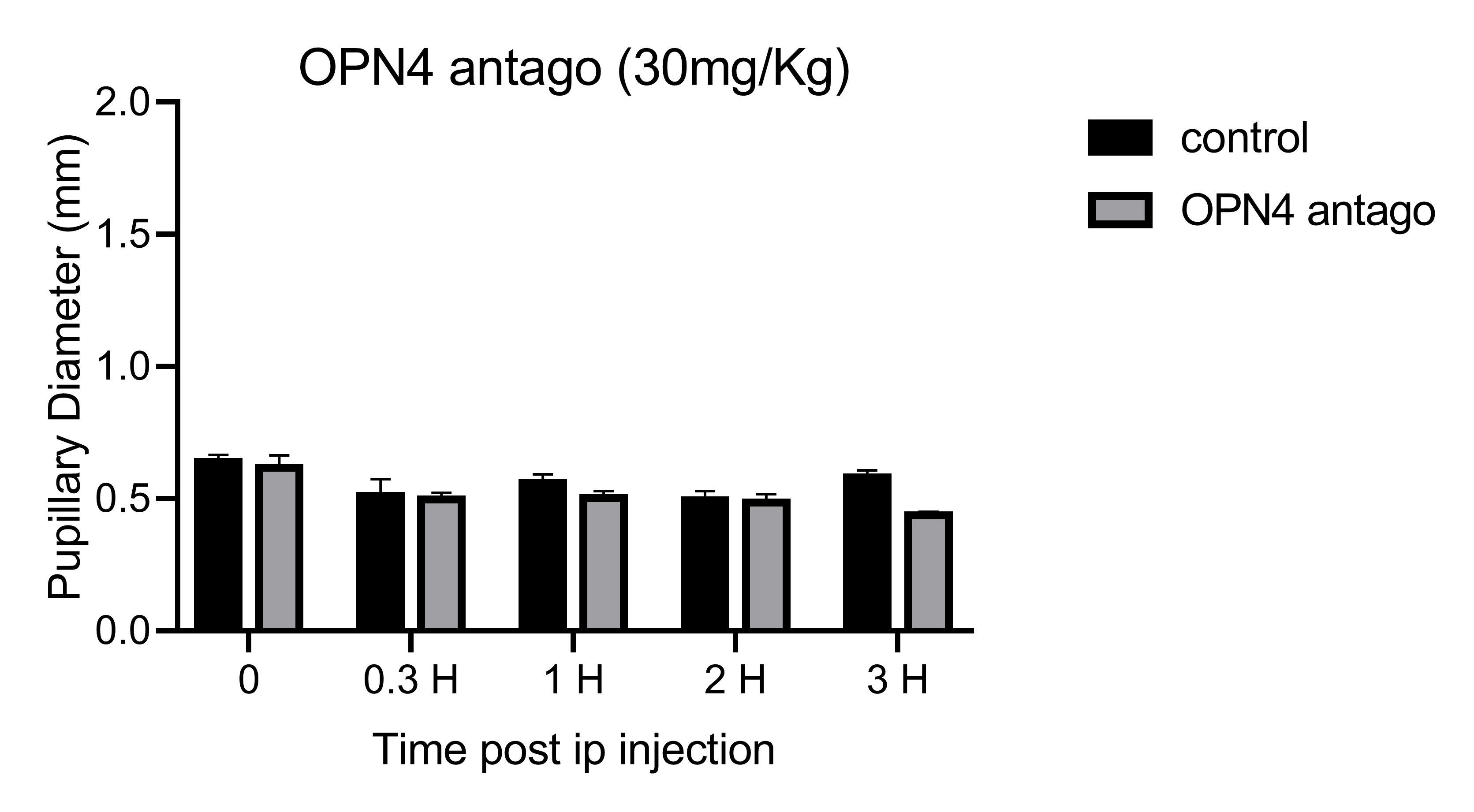

Supplement: FIGURE S3 — Opn4 antagonist action on the PLR. Measurements of the pupillary reflex at various time points in mice ip injected with the melanopsin antagonist (gray bars) as well as in the control ones (black bars). All the data are presented as mean ± SEM. No significant changes were observed. [file Image_3.TIF]

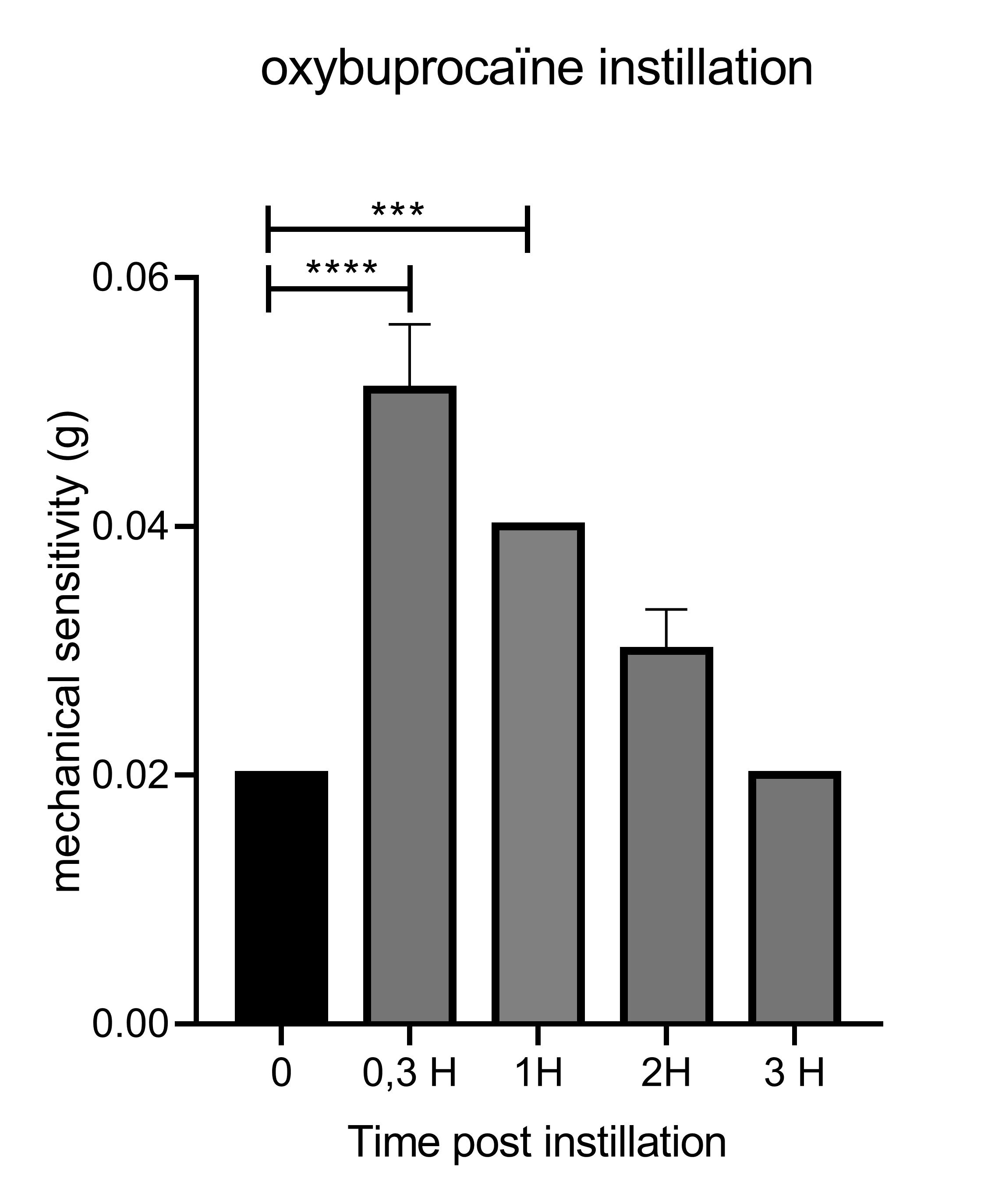

Supplement: FIGURE S4 — Corneal mechanical sensitivity after oxybuprocaine instillation. Von Frey test was realized at various time points after oxybuprocaine instillation (gray bars) and compared to the control condition (black bar). All the data are presented as mean ± SEM. [file Image_4.TIF]

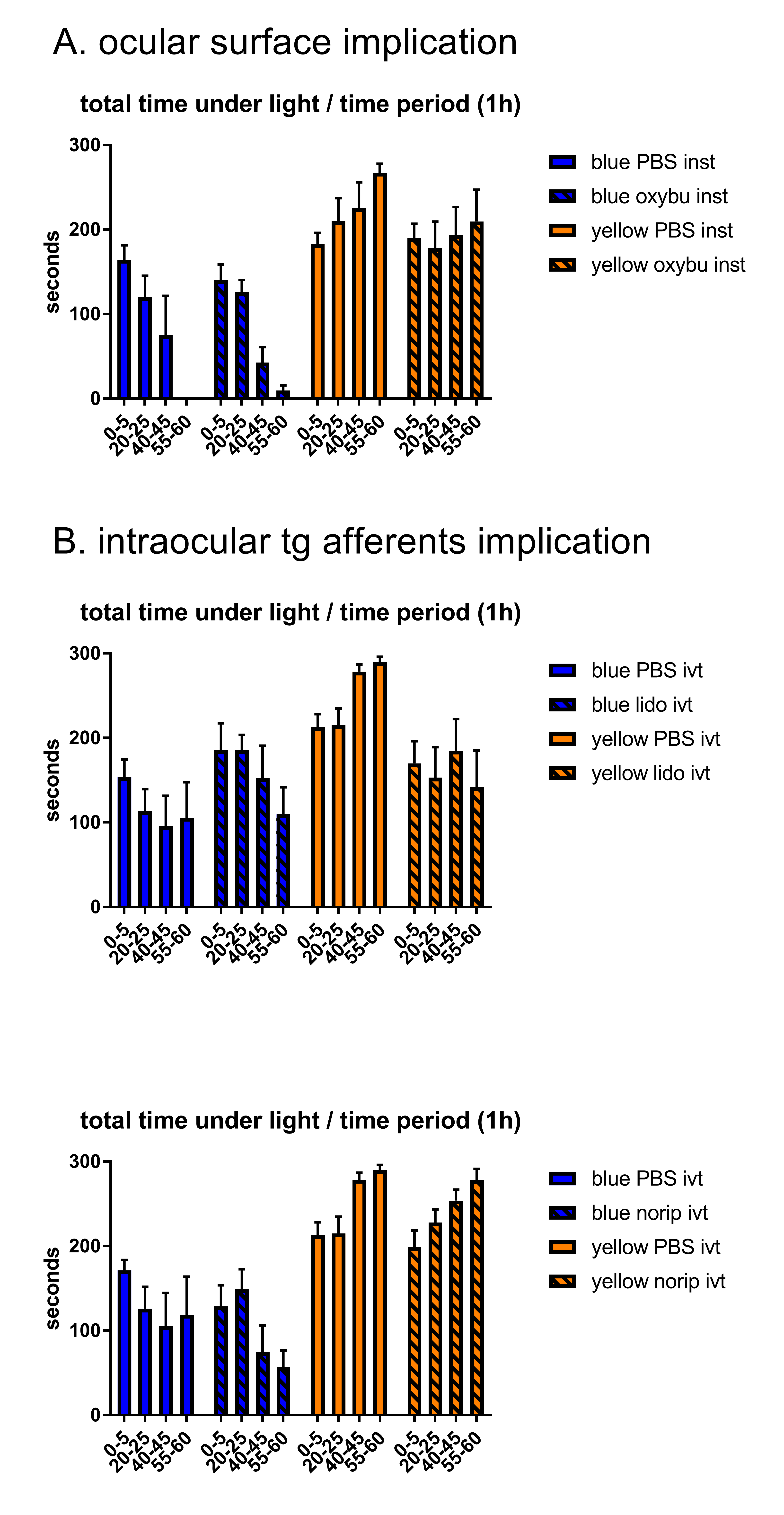

Supplement: FIGURE S5 — Shorter periods of behavioral tests. Graphs represent how the time spent in the illuminated part of the cage evolved during the 1st hour when oxibuprocaine (A), lidocaine (B) or norepinephrine (C) were applied. Numbers 0–5, 20–25, 40–45, and 55–60 (min) correspond to time periods within the 1st hour. Blue and yellow bars correspond to blue and yellow exposures respectively; clear bars and hatched bars correspond to animals with control (vehicle – PBS) or specific drug treatments respectively. All the data are presented as mean ± SEM. Stars correspond to comparisons between blue-illuminated and yellow-illuminated mice, treated with the same drug. Carets correspond to comparisons between control and drug-treated animals. Red color means increase and blue color decrease in values. [file Image_5.TIF]

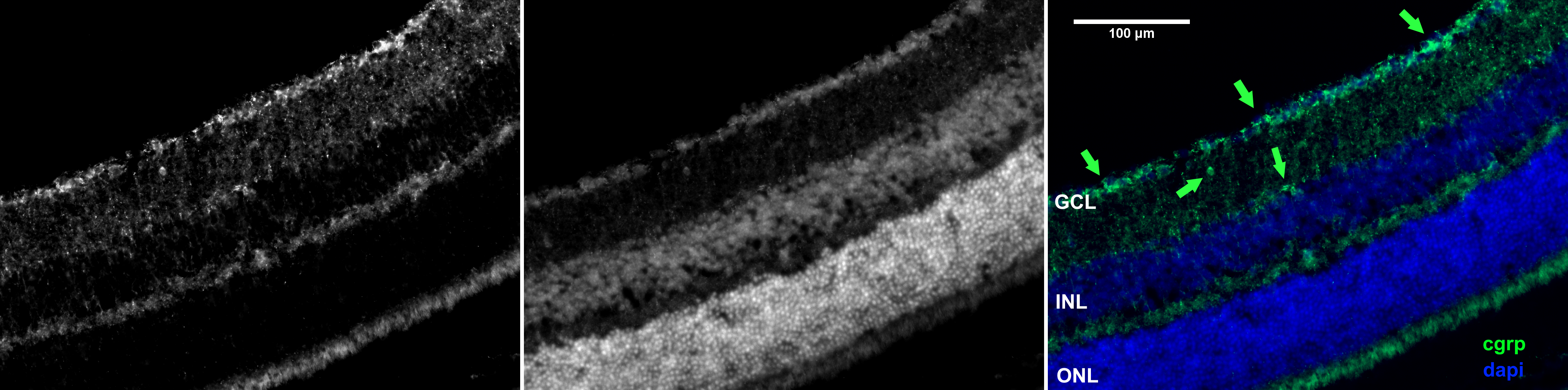

Supplement: FIGURE S6 — Potential link between the retina and TG. Immunostaining of the retina with anti-CGRP antibody. On the merged image, CGRP- and DAPI-stainings are shown in green and blue respectively; spots of specific CGRP-staining are indicated by arrows. Magnification is 20x, scale bar corresponds to 100 μm. [file Image_6.TIF]

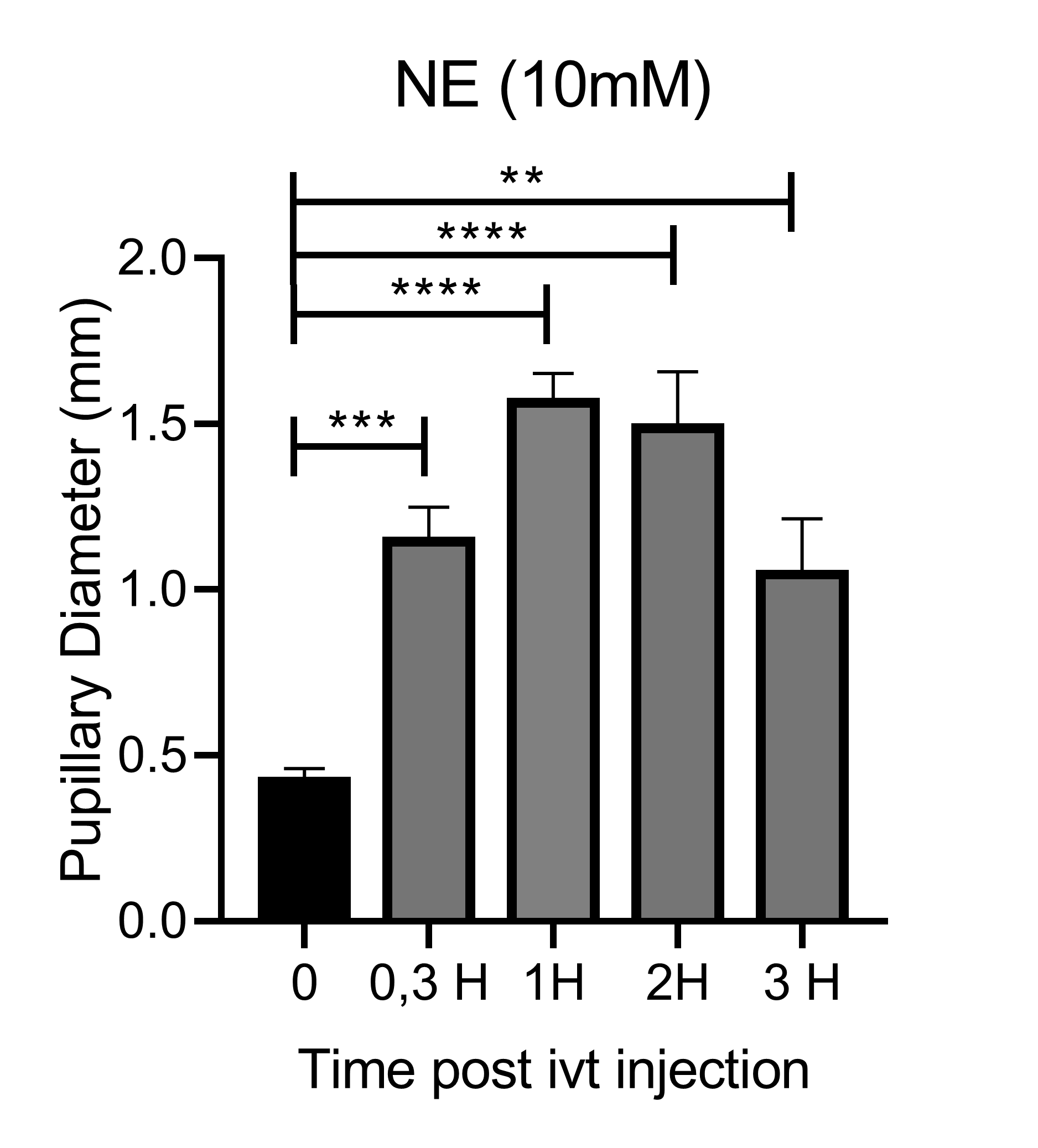

Supplement: FIGURE S7 — Mydriatic action of norepinephrine. Measurements of the pupillary reflex at various time points in mice ivt injected with norepinephrine (gray bars) as well as in the control ones (black bars). All the data are presented as mean ± SEM. [file Image_7.TIF]
